# Supplementary material for: The Bicarbonate Transporter SLC4A7 Plays a Key Role in Macrophage Phagosome Acidification
Source: Cell Host Microbe. 2018 Jun 13;23(6):766–774.e5. doi: 10.1016/j.chom.2018.04.013 (PMC6002608; doi:10.1016/j.chom.2018.04.013)
Supplement: Document S1. Figures S1 and S2 [file mmc1.pdf]

Cell Host & Microbe, Volume *23*

Supplemental Information

The Bicarbonate Transporter SLC4A7 Plays a Key Role in Macrophage Phagosome Acidification

Vitaly Sedlyarov, Ruth Eichner, Enrico Girardi, Patrick Essletzbichler, Ulrich Goldmann, Paula Nunes-Hasler, Ismet Srndic, Anna Moskovskich, Leonhard X. Heinz, Felix Kartnig, Johannes W. Bigenzahn, Manuele Rebsamen, Pavel Kovarik, Nicolas Demaurex, and Giulio Superti-Furga

Supplementary Figure 1

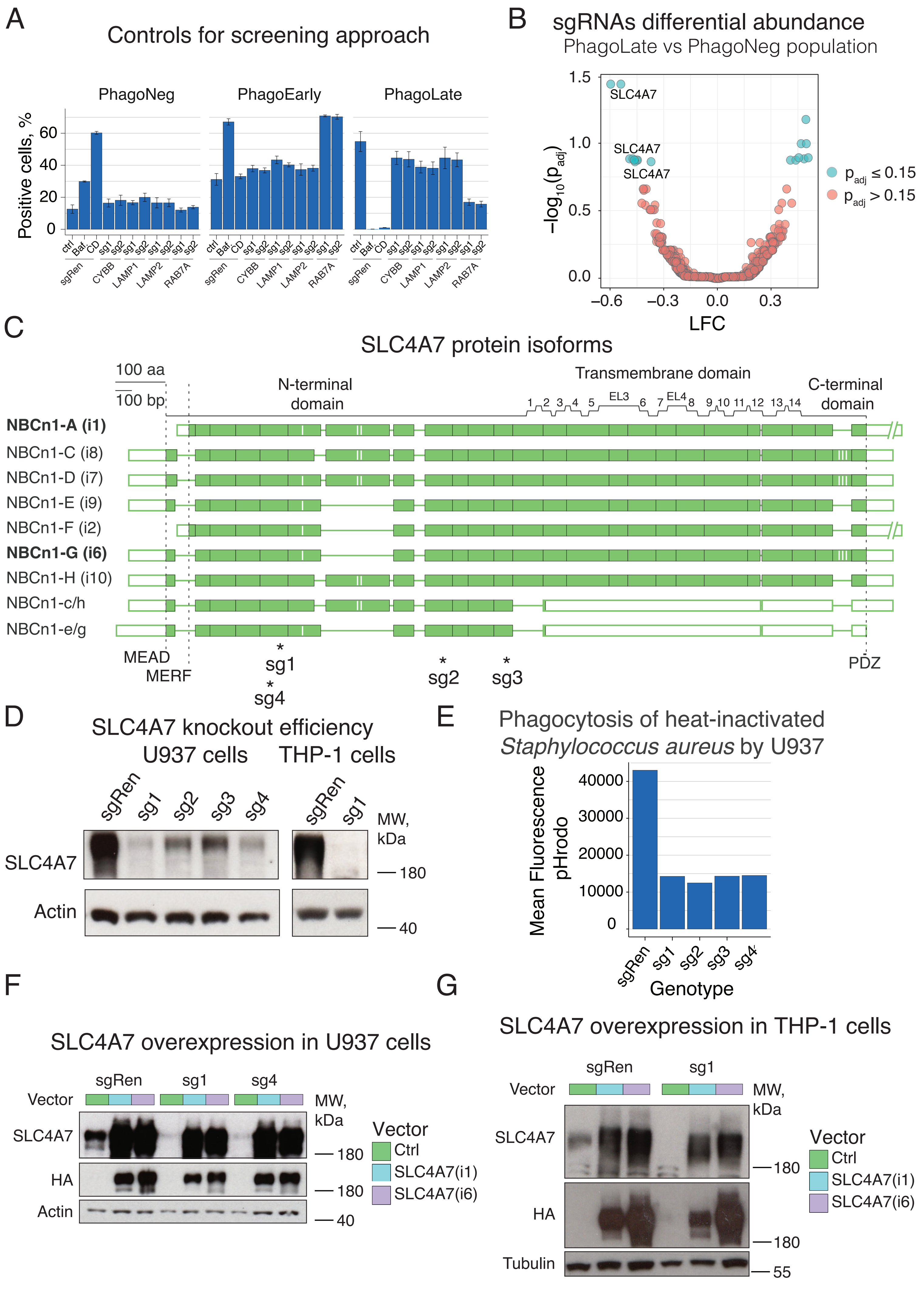

Figure S1. Loss of SLC4A7 impairs phagocytosis and phagosomal acidification. Related to Figure 1.

(A) Phagocytosis assays of U937 cells control (sgRen), treated with Bafilomycin A1 (Baf.) or Cytochalasin D (CD), NOX2 (Cybb), Lamp1, Lamp2 and Rab7a knockouts with 2 independent sgRNAs (sg1, sg2). Bar charts represent mean PhagoLate, PhagoEarly and PhagoNeg fractions as defined in Figure 1B, error bars represent 95 % confidence interval.

(B) Volcano plot showing the statistical significance of differential abundance of sgRNAs in the PhagoLate compared to the PhagoNeg fraction as $-\log_{10}(p_{adj})$ on the y-axis against their $\log_2$ fold-change (LFC) on the x-axis. The color of the dot represents statistically significant differential abundance at an estimated 15 % false discovery rate.

(C) Schematic representation of selected SLC4A7 isoforms. Exons are depicted as green rectangles and transmembrane topology is shown by the profile line above. Transmembrane domains (TMDs) are numbered 1 – 14 and the two larger extracellular loops (EL) are annotated (EL3, EL4). The alternative splicing cassettes (I – III) are shown as numbers within the exons and the two alternative start sites (“MERF” and “MEAD”) are indicated by the dotted lines. The position of the PDZ domain in the C-terminus is indicated by a dotted line. Sites targeted by the selected sgRNAs are marked with asterisks.

(D) Representative immunoblot analysis of PMA-differentiated U937 (left panel) and THP1 (right panel) cells, which were infected with the indicated sgRNA constructs (sg1-sg4) targeting SLC4A7 or control (sgRen). Lysates were probed with antibodies detecting SLC4A7 or actin as loading control.

(E) Phagocytosis assays of SLC4A7 knockout (sg1 – sg4) and control (sgRen) U937

cells with pHrodo-Red stained heat-killed *Staphylococcus aureus* USA300. Bar graphs depict mean fluorescence intensity of pHrodo signal. Increasing pHrodo-Red signal depicts increasing acidification of phagocytosed bacteria.

(F) Representative immunoblot analysis of two independent U937 clones with SLC4A7 knockout (sg1, sg4) or control (sgRen), which were infected with lentiviral expression constructs coding for Strep-HA tagged SLC4A7 isoform 1 (SLC4A7(i1)), isoform 6 (SLC4A7(i6)) or empty vector control (empty). Respective lysates were probed with the indicated antibodies.

(G) Immunoblot analysis with control (sgRen) and SLC4A7 knockout (sg1) THP-1 cells, which were infected with lentiviral expression constructs coding for Strep-HA tagged SLC4A7 isoform1 (SLC4A7-i1), isoform6 (SLC4A7-i6) or empty vector control (Ctrl). Respective lysates were probed with the indicated antibodies.

Supplementary Figure 2

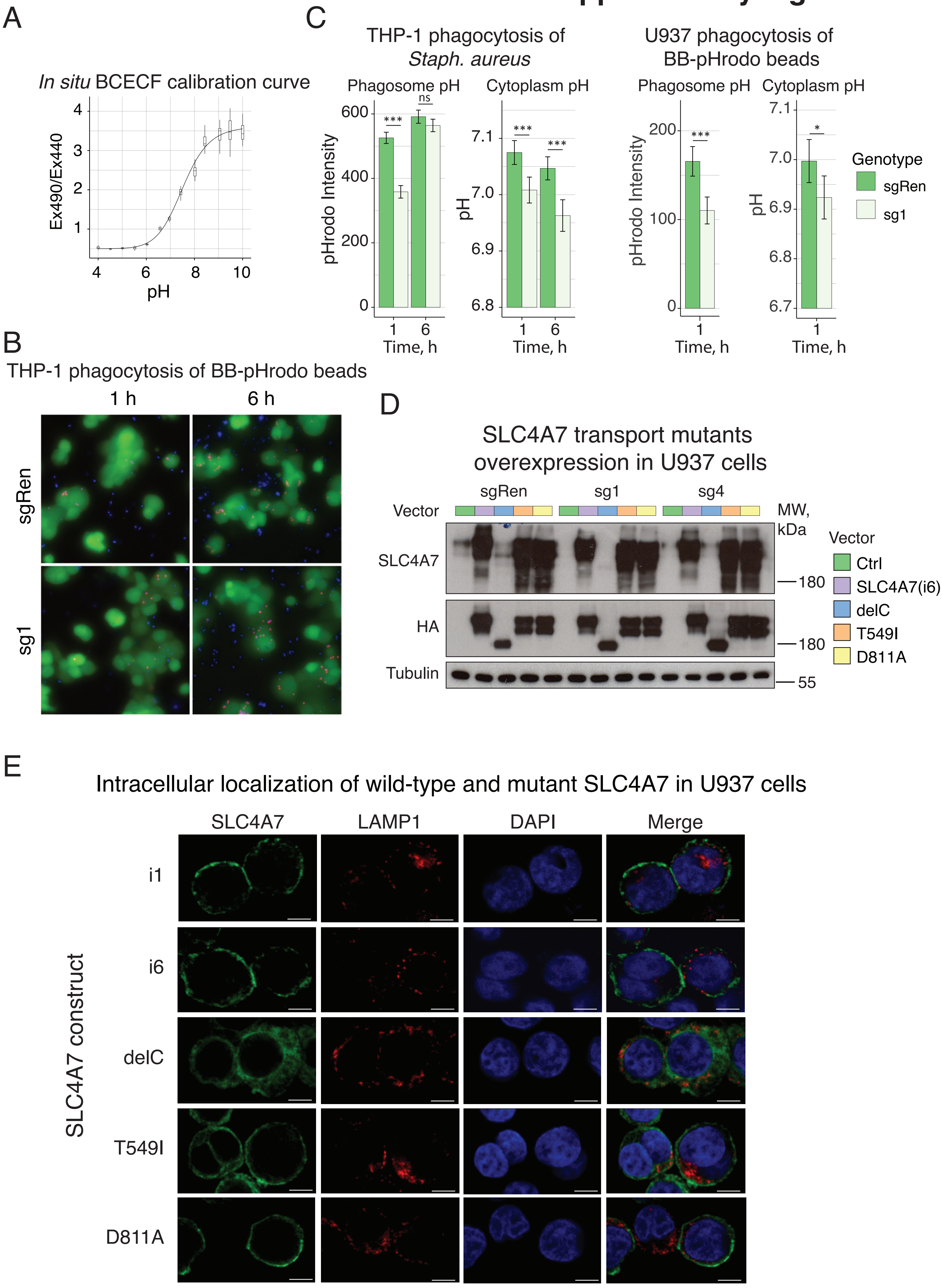

Figure S2. Reconstitution with wildtype but not mutant SLC4A7 rescues the phenotype of SLC4A7 knockout. Related to Figure 2.

(A) The *in situ* calibration curve was constructed by measuring the fluorescence ratio of BCECF emission at 525nm after excitation at 440nm or 490nm. THP-1 cells were equilibrated in potassium buffers with varying pH (4.0 – 10.0) in the presence of 10 µM nigericin and 10 µM valinomycin. Intracellular pH values are represented as box plots. Sigmoid curve was fitted to the data.

(B) Example images of simultaneous cytoplasmic and phagosomal pH measurements in THP-1 cells phagocyting bright blue pHrodo beads. THP-1 cells loaded with BCECF are shown in green (excitation 490 nm, emission 525 nm), dual-coloured (bright blue and pHrodo) beads are shown in blue and red respectively.

(C) Simultaneous measurement of cytoplasmic and phagosomal pH during phagocytosis using live-cell microscopy. Cytoplasmic and phagosomal pH of THP-1 cells loaded with BCECF-AM was measured at 1h and 6 h of pHrodo-labeled heat-killed *Staph aureus* phagocytosis (left panel). Cytoplasmic and phagosomal pH of U937 cells loaded with BCECF-AM was measured at 1h of dual-colored beads phagocytosis (right panel). Incubation and imaging were done at 37°C in 5% CO_2 in HBSS/10 % FCS buffer.

(D) Representative immunoblot analysis of two independent U937 clones with SLC4A7 knockout (sg1, sg4) or control (sgRen), which were infected with lentiviral expression constructs coding for Strep-HA tagged SLC4A7 isoform 6 (SLC4A7(i6)), a C-terminal deletion mutant (delC), two predicted transport mutants (T549I and D811A), or empty vector control (Ctrl). Lysates were probed with the indicated antibodies.

(E) Representative confocal immunofluorescence images of U937 cells infected with

lentiviral expression constructs coding for Strep-HA tagged SLC4A7 isoform 1 (i1), isoform 6 (i6), a C-terminal deletion mutant (delC) or two predicted transport mutants (T549I and D811A). Cells were differentiated with PMA and stained with antibodies to HA (green) and LAMP-1 (red). DNA counterstain with DAPI is depicted in blue. Scale bar is 5 µm.
