# Supplementary material for: The Bicarbonate Transporter SLC4A7 Plays a Key Role in Macrophage Phagosome Acidification
Source: Cell Host Microbe. 2018 Jun 13;23(6):766–774.e5. doi: 10.1016/j.chom.2018.04.013 (PMC6002608; doi:10.1016/j.chom.2018.04.013)
Supplement: Document S2. Article plus Supplemental Information [file mmc5.pdf]

Brief Report

Cell Host & Microbe

The Bicarbonate Transporter SLC4A7 Plays a Key Role in Macrophage Phagosome Acidification

Graphical Abstract

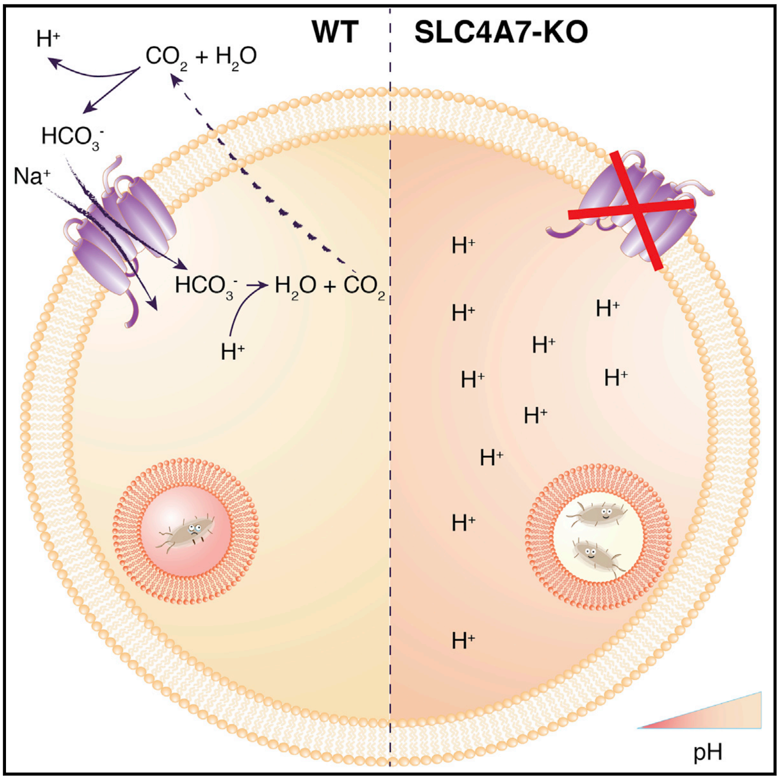

Highlights

- CRISPR screen identifies SLC4A7 to be required for phagosome acidification
- Intracellular bacterial killing by macrophages is impaired in absence of SLC4A7
- Bicarbonate transport activity of SLC4A7 is required for phagosome acidification
- SLC4A7 regulates phagosome acidification via homeostatic control of cytoplasmic pH

Authors

Vitaly Sedlyarov, Ruth Eichner, Enrico Girardi, ..., Pavel Kovarik, Nicolas Demaurex, Giulio Superti-Furga

Correspondence

gsuperti@cemm.oeaw.ac.at

In Brief

Phagosome acidification is a necessary step in pathogen clearance by macrophages. Sedlyarov et al. identify the bicarbonate transporter SLC4A7 as essential for phagosome acidification. SLC4A7-mediated bicarbonate import ensures homeostatic regulation of cytoplasmic pH. In the absence of SLC4A7, the macrophage cytoplasm acidifies, which perturbs phagosome maturation and impairs bacterial killing by macrophages.

Sedlyarov et al., 2018, Cell Host & Microbe *23*, 766–774
June 13, 2018 © 2018 The Author(s). Published by Elsevier Inc.
https://doi.org/10.1016/j.chom.2018.04.013

Cell Host & Microbe
Brief Report

The Bicarbonate Transporter SLC4A7 Plays a Key Role in Macrophage Phagosome Acidification

Vitaly Sedlyarov,[1,5] Ruth Eichner,[1,5] Enrico Girardi,[1] Patrick Essletzbichler,[1] Ulrich Goldmann,[1] Paula Nunes-Hasler,[2] Ismet Srndic,[1] Anna Moskovskich,[1] Leonhard X. Heinz,[1] Felix Kartnig,[1] Johannes W. Bigenzahn,[1] Manuele Rebsamen,[1] Pavel Kovarik,[3] Nicolas Demaurex,[2] and Giulio Superti-Furga[1,4,6,*]
[1]CeMM Research Center for Molecular Medicine of the Austrian Academy of Sciences, Vienna 1090, Austria
[2]Department of Cell Physiology and Metabolism, University of Geneva, Geneva 1211, Switzerland
[3]Max F. Perutz Laboratories, University of Vienna, Vienna Biocenter (VBC), Vienna 1030, Austria
[4]Center for Physiology and Pharmacology, Medical University of Vienna, Vienna 1090, Austria
[5]These authors contributed equally
[6]Lead Contact
*Correspondence: gsuperti@cemm.oeaw.ac.at
https://doi.org/10.1016/j.chom.2018.04.013

SUMMARY

Macrophages represent the first line of immune defense against pathogens, and phagosome acidification is a necessary step in pathogen clearance. Here, we identified the bicarbonate transporter SLC4A7, which is strongly induced upon macrophage differentiation, as critical for phagosome acidification. Loss of SLC4A7 reduced acidification of phagocytosed beads or bacteria and impaired the intracellular microbicidal capacity in human macrophage cell lines. The phenotype was rescued by wild-type SLC4A7, but not by SLC4A7 mutants, affecting transport capacity or cell surface localization. Loss of SLC4A7 resulted in increased cytoplasmic acidification during phagocytosis, suggesting that SLC4A7-mediated, bicarbonate-driven maintenance of cytoplasmic pH is necessary for phagosome acidification. Altogether, we identify SLC4A7 and bicarbonate-driven cytoplasmic pH homeostasis as an important element of phagocytosis and the associated microbicidal functions in macrophages.

Cellular metabolism is central to many specific functions of immune cells. Accordingly, the interplay between metabolism and specific functions of immune cells has emerged as key focus in immunological research (Buck et al., 2017). Macrophages represent the first line of immune defense against pathogens. Upon activation, they undergo massive metabolic reprogramming to allow execution of specialized programs such as phagocytosis (Artyomov et al., 2016; O'Neill and Pearce, 2016; Shapiro et al., 2011). An appropriate metabolic response requires the cell to rapidly interact with its environment for nutrient uptake and ion transport (Lin et al., 2015; Sahoo et al., 2014). Movement of metabolites and molecules across cellular membranes is mainly achieved by transporter proteins, such as solute carriers (SLCs), which represent the largest group of facilitative and concentrative transporters in the human genome (César-Razquin et al., 2015).

We hypothesized that SLCs could be essential for macrophages to undergo the metabolic changes associated with phagocytosis, and aimed for an appropriate model system suitable for CRISPR/Cas9-based genetic screens (Figure 1A).

Phagocytosis and phagosome maturation eventually lead to progressive acidification of phagolysosomes (Kinchen and Ravichandran, 2008). Phagosome acidification, detected by pH-dependent fluorophores is a valid surrogate endpoint to measure phagocytosis (Colas et al., 2014; Fine et al., 2017; Nunes et al., 2015; Surewaard and Kubes, 2017; Takahashi et al., 2017). We chose phorbol myristate acetate (PMA)-differentiated human myeloid U937 cells as a macrophage model system to detect and quantify phagosome acidification (Liu and Wu, 1992). We subjected differentiated U937 cells to phagocytosis assays with opsonized latex beads, which were coupled to a pH-sensitive (pHrodo) and a pH-insensitive dye (YG, yellow green) (Figure 1B) (Colas et al., 2014). The dual-colored beads allowed discrimination between cells that underwent phagocytosis and phagosome acidification (PhagoLate, YG and pHrodo-positive), cells that have bound and/or phagocytosed, but not acidified the cargo (PhagoEarly, only YG-positive), and cells that have neither bound nor incorporated any beads (PhagoNeg) (Figure 1B).

To validate our experimental strategy, we tested a range of pharmacological and genetic perturbations targeting different stages of the phagocytic process (Figure S1A). Disruption of phagocytic uptake using the actin polymerization inhibitor cytochalasin D led to ablation of the PhagoLate fraction with simultaneous increase of the PhagoNeg, but not the PhagoEarly fraction. By contrast, inhibition of phagosome acidification using the vacuolar-ATPase inhibitor bafilomycin A1 resulted in a strong decrease of the PhagoLate fraction, accompanied by an elevation of the PhagoEarly and, to lesser extent, of the PhagoNeg fraction. CRISPR-mediated knockout of nicotinamide adenine dinucleotide phosphate (NADPH) oxidase 2 (NOX2 or CYBB), a major producer of reactive oxygen species during phagocytosis, and of Lamp1 or Lamp2, important lysosomal structural proteins, resulted in a slight decrease of the PhagoLate and minor increases in the PhagoEarly and PhagoNeg fractions, corresponding to a slight decrease in phagosome acidification (Figure S1A). Finally, knockout of RAB7A, a small guanosine

 © 2018 The Author(s). Published by Elsevier Inc.
This is an open access article under the CC BY license (http://creativecommons.org/licenses/by/4.0/).

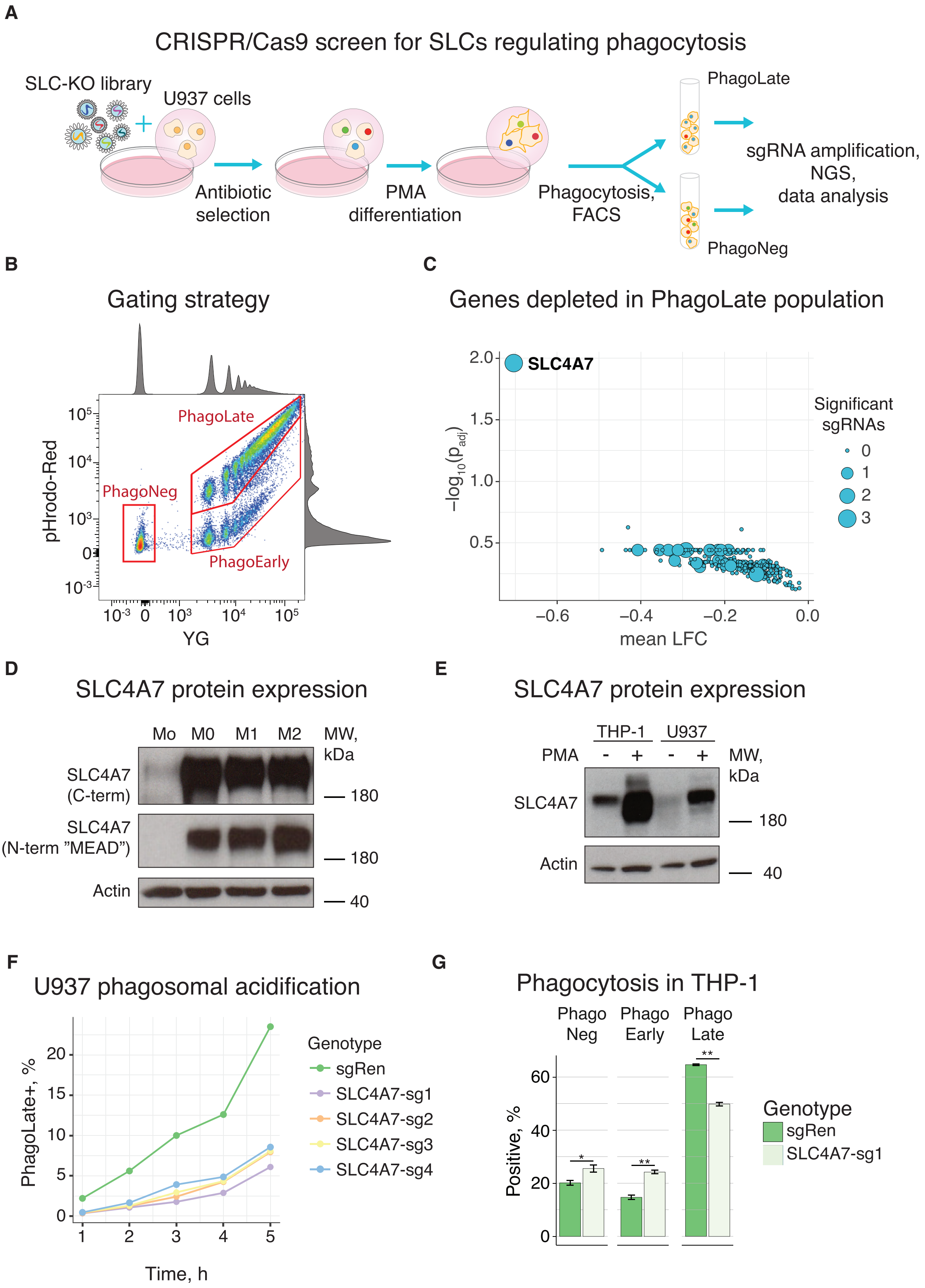

(legend on next page)

triphosphatase required for phagosome maturation and phagosome-to-lysosome fusion, strongly reduced the PhagoLate fractions, while strongly increasing the PhagoEarly, but not the PhagoNeg fractions, corresponding to a strong inhibition of phagosome maturation (Soldati et al., 1995; Via et al., 1997) (Figure S1A).

Having validated the experimental strategy, we set out to systematically identify SLCs involved in the regulation of phagocytosis. We established a CRISPR/Cas9-based loss-of-function screen using an SLC-wide knockout library, designed in our laboratory, and a fluorescence-activated cell sorting (FACS)-based readout. The pooled library targets 391 human SLC genes with six single guide RNAs (sgRNAs) per gene and additionally comprises sgRNAs targeting essential genes and non-targeting sgRNAs as controls. We infected U937 cells with lentiviral particles carrying the SLC knockout library and differentiated them with PMA to establish a macrophage-like phenotype (Figure 1A). To identify SLCs essential for phagocytosis, we isolated two populations via FACS: the phagocytosis- and acidification-positive fraction (PhagoLate) and the phagocytosis-negative population (PhagoNeg) (Figure 1B). After amplification and sequencing of the sgRNAs from each population, the reads were mapped and quantified using a two-step differential abundance analysis. After identifying differentially enriched sgRNAs using DESeq2 (Figure S1B; Data S1) (Love et al., 2014), we aggregated sgRNAs to genes using the gene set enrichment algorithm (Figure 1C) (Sergushichev, 2016; Subramanian et al., 2005). Strikingly, among all SLCs, SLC4A7 was the only gene significantly depleted from the PhagoLate fraction (Figures 1C and S1B).

SLC4A7, also known as NBCn1 and NBC3, is an electroneutral sodium bicarbonate co-transporter involved in maintenance of cytosolic pH by extruding acid equivalents (Lee et al., 2014; Romero et al., 2013). SLC4A7 has a high number of different isoforms, which show tissue-specific expression and differ in surface abundance and transport activity. They arise from two alternative promotors, three optional structural elements (cassettes I-III), and respective alternative splicing (Figure S1C) (Liu et al., 2013; Parker and Boron, 2013; Pushkin et al., 1999). SLC4A7 is expressed in several cell types and tissues including brain, heart, and kidney (Damkier et al., 2007; Liu et al., 2015; Pushkin et al., 1999; Romero et al., 2013). Although data on SLC4A7 expression in the immune system are scarce, there is evidence for a specific role in macrophages. SLC4A7 was shown to be strongly upregulated upon macrophage colony-stimulating factor (M-CSF)- and receptor activator of nuclear factor-κB ligand-induced osteoclast differentiation (Riihonen et al., 2010). Moreover, a transcriptional study identified SLC4A7 among the genes differentially expressed during the monocyte-to-macrophage differentiation and polarization (Martinez et al., 2006). To further investigate the potential specific role of SLC4A7 in macrophages, we treated primary human monocytes with M-CSF and observed a strong upregulation of SLC4A7 upon macrophage differentiation, suggesting a particular function in macrophage biology (Figure 1D). Polarization of macrophages to the M1 phenotype or the M2 phenotype did not further modulate SLC4A7 protein abundance (Figure 1D). Consistently, the expression levels of SLC4A7 in human U937 and THP-1 cells strongly increased upon PMA-induced differentiation toward macrophages (Figure 1E).

To validate the findings of the CRISPR screen, we generated SLC4A7-deficient U937 cells using four different sgRNAs targeting different exons common to all SLC4A7 isoforms (Figures S1C and S1D). After confirming SLC4A7 knockout efficiency by immunoblot (Figure S1D), we performed phagocytosis assays with the respective U937 cells at different time points. Importantly, at each time point, all four sgRNAs resulted in strongly decreased numbers of PhagoLate cells as compared with control cells infected with a non-targeting sgRNA (sgRen, directed toward *Renilla* luciferase) (Figure 1F).

If SLC4A7 plays a fundamental role in phagocytosis, it should do so also in other human macrophage model cell lines. We CRISPR/Cas9-inactivated SLC4A7 in human THP-1 myeloid cells and differentiated them with PMA. Phagocytosis assays showed a significant reduction in the PhagoLate fraction upon SLC4A7 knockout, which was accompanied by an increase in

Figure 1. SLC-Focused CRISPR/Cas9 Genetic Loss-of-Function Screens Identify SLC4A7 to Be Important for Phagosome Acidification
(A) Schematic representation of the major steps of the SLC-focused CRISPR/Cas9 screen to identify SLCs involved in phagocytosis.
(B) Representative flow cytometry scatterplot of phagocytosis assays. PMA-differentiated U937 cells were incubated with dual-colored opsonized beads. Each dot represents one cell: intensity of the pH-insensitive dye (YG) is displayed on the x axis, intensity of pH-sensitive dye (pHrodo-Red), whose signal intensity increases with decrease in pH, is shown on the y axis. Double-negative cells were classified as phagocytosis-negative (PhagoNeg), double-positive cells (YG and high pHrodo-Red signal) were classified as cells having undergone phagocytosis and phagosome acidification (PhagoLate), and single positive cells (YG and low pHrodo-Red signal) were classified as cells at early stages of phagocytosis (PhagoEarly). The marginal intensity distributions are shown on the sides of the plot.
(C) Volcano plot showing the statistical significance of genes depleted in the PhagoLate population on the y axis as $-\log_{10}(p_{adj})$ against average $\log_2$ fold-change (mean LFC) on the x axis calculated for all sgRNAs per gene. The size of the dots represents the number of significant changes in sgRNAs counts. See Figure S1B for differential abundance of individual sgRNAs.
(D) Immunoblot analysis of primary human monocytes (Mo) derived from peripheral blood and M-CSF-differentiated monocyte-derived human macrophages, which were unpolarized (M0), or polarized toward M1 phenotype with interferon-γ and lipopolysaccharide, or to M2 phenotype with interleukin-4. Respective lysates were probed with an anti-SLC4A7 antibody detecting all isoforms (C-terminal epitope) and an anti-SLC4A7 antibody detecting an N-terminal epitope present only in isoforms starting with the amino acids "MEAD." Actin was used as loading control. MW, molecular weight. See Figure S1C for isoforms.
(E) Representative immunoblot analysis of SLC4A7 expression in undifferentiated and PMA-differentiated THP-1 and U937 cells. Actin was used as loading control. MW, molecular weight.
(F) Representative kinetics of phagosome acidification in four independent SLC4A7 knockout (sg1-sg4) and control (sgRen) U937 cells. After PMA differentiation, U937 cells were incubated with dual-colored beads and analyzed at the indicated time points using flow cytometry. The fraction of PhagoLate cells is displayed against the incubation time with beads. See Figure S1D for confirmation of SLC4A7 knockdown.
(G) Phagocytosis assays with PMA-differentiated SLC4A7 knockout (sg1) or control (sgRen) THP-1 cells, which were incubated with dual-colored beads as described in (B). Bar graphs show the fraction of PhagoLate, PhagoEarly, and PhagoNeg cells as assessed by pHrodo and YG fluorescence in flow cytometry. Data are mean ± 95% confidence interval from four replicates. $^*p < 0.05$, $^{**}p < 0.001$; by Welsh's t test.

the PhagoEarly and, to a minor extent, of the PhagoNeg fraction (Figure 1G). This pattern was comparable with the phenotype of hampered phagosome acidification (Figure S1A). Therefore, the reduced number of PhagoLate cells was assumed to be the main effect, with the changes in the other fractions being secondary phenomena.

Together, the data demonstrate the general importance of SLC4A7 for phagosome acidification. To test the relevance of these findings for host-pathogen interactions, we subjected SLC4A7 knockout and control U937 cells to phagocytosis assays with pHrodo-labeled heat-inactivated *Staphylococcus aureus*. In analogy to labeled beads, we witnessed a strong decrease in the capacity of SLC4A7 knockout cells for acidification of phagocytosed bacteria (Figure S1E), suggesting that SLC4A7 is important for phagosome acidification of pathogens.

To test the specificity of the phenotype, we undertook reconstitution experiments. Among the different isoforms of SLC4A7, we chose two representative forms: isoform 1 (NBCn1-A), the canonical form, which is mainly expressed in cardiac and skeletal muscle, and isoform 6 (NBCn1-G), the most common form, which is expressed in various tissues and differs from isoform 1 in both N terminus and two splicing cassettes (Figure S1C) (Cooper et al., 2006; Liu et al., 2013). U937 sgRen and sgSLC4A7 cells were successfully reconstituted with lentiviral overexpression constructs coding for isoform 1 or 6 of SLC4A7, as confirmed by immunoblotting (Figure S1F). Overexpression of both isoforms increased the PhagoLate cell fraction beyond the mere rescue of deficient phagosome acidification (Figure 2A). Markedly, in U937, isoform 6 appeared to be more potent in increasing phagosome acidification than isoform 1, which could result from its reported higher bicarbonate transport and pH-buffering capacity (Liu et al., 2013). Interestingly, an antibody directed against the N-terminal sequence containing the amino acids "MEAD," present in isoform 6, but not 1 (Figure S1C), confirmed that MEAD-forms of SLC4A7 were induced in macrophages, and hence are of physiological relevance (Figure 1D). In THP-1 cells, overexpression of both SLC4A7 isoforms also increased the degree of phagosome acidification beyond the mere rescue of the sgSLC4A7 phenotype (Figures 2B and S1G). In this cellular context, however, there was no difference between isoform 1 and 6, which may result from the higher baseline phagocytic rate and saturation of the system (Figure 2B).

The microbicidal activity of phagosomes results from direct bacterial killing in an acidic environment and activation of pH-sensitive antimicrobial enzymes (Flannagan et al., 2015). To test if impaired phagosome acidification upon SLC4A7 loss decreased the intracellular bacterial killing capacity, we performed intracellular bacterial killing assays and infected PMA-differentiated THP-1 cells with Gram-negative (*Escherichia coli* K12) and Gram-positive (*Streptococcus pyogenes* ΔSLO strain and *Staphylococcus carnosus*) bacteria. Importantly, THP-1 cells deficient for SLC4A7 showed a significantly reduced intracellular killing capacity toward *E. coli*, *S. pyogenes*, and *S. carnosus*, compared with control cells (Figure 2C, left panel). Upon reconstitution of knockout cells with SLC4A7 isoform 6, the phenotype was rescued and normal intracellular bactericidal capacity restored (Figure 2C, left panel). Next, we performed intracellular killing assays with the *S. aureus* strains Newman and USA300, which both stem from clinical isolates. While Newman is pH sensitive, USA300 depends on phagosome acidification for intracellular survival and proliferation within macrophages (Tranchemontagne et al., 2016). In line with previous results, SLC4A7-deficient THP-1 cells displayed a reduced killing capacity toward the *S. aureus* Newman strain. In contrast, killing of the *S. aureus* USA300 strain was increased in the knockout cells compared with control (Figure 2C, right panel), suggesting impaired intracellular survival due to reduced acidification. Taken together, these data provide strong evidence for the importance of SLC4A7 in efficient phagosome acidification and microbicidal potency of the cells.

Given its role in bicarbonate transport and pH regulation, and the evidence that SLC4A7 isoforms with distinct bicarbonate transport capacity differentially affected phagosome acidification (Figure 2B), it can be concluded that SLC4A7-mediated bicarbonate transport is essential for proper phagosome acidification. If located at phagosomal membranes, SLC4A7 could theoretically affect phagosomal pH directly. By contrast, if localized exclusively at the plasma membrane, the mechanism would likely be indirect via regulation of cytoplasmic pH. Visualization of endogenous SLC4A7 in PMA-differentiated THP-1 cells using indirect immunofluorescence revealed a predominant localization of SLC4A7 at the plasma membrane (Figure 2D), which is in line with previous reports (Loiselle et al., 2003; Wang et al., 2015a). To investigate the dynamic localization of SLC4A7 during phagocytosis, we expressed GFP-tagged SLC4A7 isoform 6 in THP-1 cells and exposed them to pHrodo-labeled heat-inactivated *S. aureus* or dual-colored (pHrodo and bright blue) beads. Live-cell microscopy including time-lapse imaging confirmed the cell surface localization of SLC4A7 (Figure 2E; Video S1). Importantly, no association of SLC4A7 with the phagosomal cargo could be observed during the phagocytic process (Figure 2E; Video S1).

To test the hypothesis that SLC4A7 regulates phagosome acidification indirectly via dynamic cytoplasmic pH regulation, we undertook live-cell microscopy-based experiments. Using the ratiometric cytoplasmic pH indicator, BCECF-AM, and pHrodo-coated bacteria and beads, we simultaneously measured cytosolic and phagosomal pH at different time points during the phagocytic process (Figures S2A and S2B). At 1 hr after onset of phagocytosis of beads, neither average pHrodo intensity nor cytoplasmic pH differed between SLC4A7 knockout and control THP-1 cells (Figure 2F). At 6 hr, however, with increasing phagosome acidification, differences became apparent: while the phagosomal cargos were less acidified in SLC4A7 knockout cells, the cytoplasm was significantly more acidic (Figures 2F and S2C). Reduced phagosome acidification accompanied by more acidic cytoplasmic pH upon SLC4A7 knockout was consistently observed across different cell lines (THP-1 and U937) and phagosomal cargos (beads and heat-killed *S.aureus*) (Figures 2F and S2C).

Together with its specific upregulation upon macrophage differentiation, these findings argue against a general housekeeping role of SLC4A7 in cytosolic pH homeostasis. Instead, SLC4A7 seems to be of particular relevance for counteracting cytoplasmic acidification during the phagocytic process.

We next wanted to investigate whether bicarbonate transport was essential for SLC4A7-mediated pH regulation. As the 3D

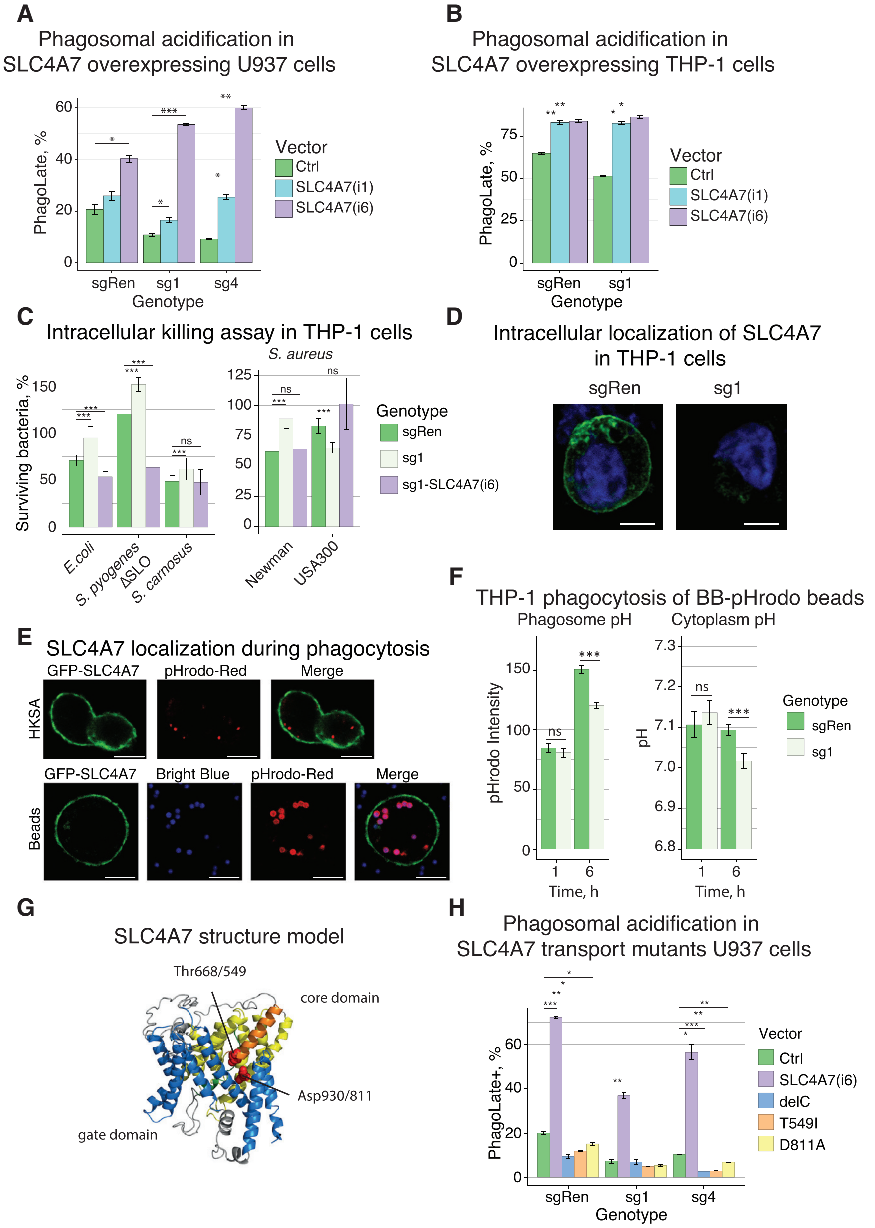

Figure 2. Functional Consequences of SLC4A7 Knockout and Overexpression

(A) Phagocytosis assays with control (sgRen) and SLC4A7 knockout (sg1, sg4) U937 cells, which were lentivirally infected to exogenously express HA-tagged SLC4A7 isoform 1, isoform 6, or empty vector control (Ctrl), respectively. Cells were incubated with dual-colored beads as described in Figure 1B. Bar graphs show the fraction of PhagoLate, PhagoEarly, and PhagoNeg cells as assessed by flow cytometry. Data are mean ± 95% confidence interval from three replicates. *$p < 0.05$, **$p < 0.01$, ***$p < 0.001$; by Welsh's t test. See Figure S1F for immunoblot confirmation of SLC4A7 knockout and exogenous expression.

(B) Phagocytosis assays with control (sgRen) and SLC4A7 knockout (sg1) THP-1 cells, which were lentivirally infected to exogenously express HA-tagged SLC4A7 isoform 1, isoform 6, or empty vector control (Ctrl), respectively. Cells were incubated with dual-colored beads as in Figures 1B and 2A. Bar graphs show the fraction of PhagoLate, PhagoEarly, and PhagoNeg cells as assessed by flow cytometry. Data are mean ± SD from two replicates. *$p < 0.05$, **$p < 0.01$; by Welsh's t test. See Figure S1G for immunoblot confirmation of SLC4A7 knockdown and exogenous expression.

(C) Intracellular killing assay with viable Gram-negative (*E. coli*) and Gram-positive (*Streptococcus pyogenes* ΔSLO, *Staphylococcus carnosus* Schleifer and Fischer, and *Staphylococcus aureus* Newman and USA300) bacteria in control (sgRen), SLC4A7 knockout (sg1), and SLC4A7 knockout reconstituted with SLC4A7 isoform 6 (sg1-SLC4A7(i6)) THP-1 cells. Bar graphs depict the percentage of surviving intracellular bacteria in relation to time point zero. Data are median and interquartile range from three replicates. ns, not significant, ***$p < 0.001$; by Wilcoxon-Mann-Whitney test.

(D) Representative confocal immunofluorescence images of endogenous SLC4A7 in control (sgRen) or SLC4A7 knockout (sg1) THP-1 cells. PMA-differentiated cells were fixed and stained with anti-SLC4A7 antibody (green). DNA was counterstained with DAPI (blue). The overlay of both signals is depicted. Scale bars, 5 μm.

(E) Representative confocal live-cell immunofluorescence images of THP-1 cells expressing GFP-tagged SLC4A7 isoform 6. After PMA-induced differentiation, cells were incubated with pHrodo-labeled heat-killed *S. aureus* (HKSA, upper panel) or dual-colored beads (pHrodo and bright blue; lower panel). Single channel images and respective overlays are shown. Scale bars, 10 μm. For time-lapse acquisitions, see Video S1.

(F) Simultaneous measurement of cytoplasmic and phagosomal pH during phagocytosis using live-cell microscopy. PMA-differentiated control (sgRen) and SLC4A7 knockout (sg1) THP-1 cells were loaded with BCECF-AM, incubated with dual-colored beads (pHrodo and bright blue), and imaged at the indicated time points. Incubation and imaging were done in Hank's balanced salt solution with 10% FCS at 37°C in 5% CO_2. At each time point, z stacks of five different fields were acquired per replicate. Bar charts represent pHrodo intensities of phagocytosed beads or cytoplasmic pH as calculated based on the BCECF calibration curve. Data are mean and 95% confidence interval from three replicates. ***$p < 0.001$; by Welch's t test. For *in situ* calibration of the BCECF 490/440 ratio, see Figure S2A; for example images, see Figure S2B. For simultaneous cytoplasmic and phagosomal pH measurements in THP-1 cells phagocyting heat-killed *S. aureus*, see Figure S2C (left panel), and for U937 cells phagocytosing beads, see Figure S2C (right panel).

(G) Schematic representation of the SLC4A7 model with the transmembrane domains (TMDs) of the core domain in yellow and the TMDs of the gate domain in blue. Helix 3 and helix 10, which align to form a continuous helix near the putative substrate binding site, are shown in orange and green, respectively. The residues mutated in the functional studies are shown in red with the isoform1/isoform6 numbering scheme.

(H) Phagocytosis assays with two independent U937 clones with SLC4A7 knockout (sg1, sg4) or control (sgRen), which were infected with lentiviral expression constructs coding for Strep-HA-tagged SLC4A7 isoform 6 (SLC4A7(i6)), an isoform 6 mutant lacking amino acids 1,008–1,131 (delC), two different predicted transport mutants (T549I and D811A), or empty vector control (Ctrl). PMA-differentiated cells were incubated with dual-colored beads as in Figure 1B and analyzed by flow cytometry. Bar graphs show the fraction of PhagoLate cells as assessed by pHrodo fluorescence intensity. Data are mean and 95% confidence interval from two replicates. *$p < 0.05$, **$p < 0.01$, ***$p < 0.001$; by Welsh's t test. For subcellular localization of the different proteins, see immunofluorescence analysis in Figure S2E.

structure of SLC4A7 is unknown, we generated a model of the transmembrane region of SLC4A7 with the I-Tasser Suite (Yang et al., 2015), using the available SLC4A1 structure as a template (PDB: 4YZF; Arakawa et al., 2015). The C-terminal domain of SLC4A7 shares 42% sequence identity with the corresponding domain of SLC4A1, and the model closely resembles the overall SLC4A1 architecture (Figure 2G). Mapping of functionally important residues on our model identified Asp930 (Asp811 in isoform 6) and Thr668 (Thr549 in isoform 6) as positions likely to be required for transporter activity (Figure 2G). Asp930 corresponds to SLC4A1 Glu681, a residue critical for anion exchange, while Thr668 corresponds to Ser465 in SLC4A1, a position tolerating only conservative mutations (Chernova et al., 1997; Barneaud-Rocca et al., 2013; Bonar et al., 2013). We generated mutant versions of SLC4A7 isoform 6 carrying the Asp811Ala (D811A) or the Thr549Ile (T549I) point mutation (Figure 2G). In addition, we generated a deletion mutant lacking the cytoplasmic C-terminal domain of SLC4A7, which contains a PDZ domain and is essential for membrane localization and function of SLC4A7 (Loiselle et al., 2003). We used all three mutants to reconstitute U937 cells lacking endogenous SLC4A7. Importantly, both the D811A and T549I transport mutants showed expression levels and subcellular localization comparable with the wild-type protein, whereas the C-terminal deletion mutant failed to localize to the plasma membrane (Figures S2D and S2E) (Loiselle et al., 2003). Notably, neither wild-type nor mutated SLC4A7 co-localized with the lysosomal marker Lamp1 (Figure S2E). In contrast to the wild-type protein, none of the three SLC4A7 mutants was able to rescue the phenotype of SLC4A7 knockout (Figure 2H). Whereas failure of the deletion mutant to rescue could be explained by its mislocalization (Figure S2E), the inability of both point mutants to rescue phagosome acidification suggested that transporter activity is essential for the biological function of SLC4A7. This supports the assumption that bicarbonate import into the cytosol is essential for cytosolic pH homeostasis and efficient phagosome acidification.

How Do We Envisage the Role of SLC4A7 in the Regulation of Phagocytosis?

The process of phagocytosis is associated with a rapid and transient acidification of the cytoplasm, which is believed to result from both respiratory burst and increased metabolic acid production (Coakley et al., 2002; Grinstein et al., 1991; Morgan et al., 2009; Swallow et al., 1991). Failure to counterbalance the cytoplasmic acidification results in cytoplasmic hyper-acidification, which, among others, inhibits the NADPH oxidase and reduces phagosome acidification (Coakley et al., 2002; Morgan et al., 2009). So far, it was assumed that Na^{+}/H^{+} exchangers, passive proton conductance channels, and voltage-gated proton channels are the main players responsible for counteracting the initial cytoplasmic acidification (Coakley et al., 2002; Morgan et al., 2009). However, many studies were undertaken in nominally bicarbonate-free buffers, suggesting that the impact of bicarbonate transporters might have been overlooked.

The strong induction of SLC4A7 during macrophage differentiation, and the failure to maintain cytoplasmic pH during phagocytosis upon genetic inactivation, suggest SLC4A7 to be of key importance for pH homeostasis in phagocyting macrophages. Cell surface localization of SLC4A7 and the inability of transport mutants to maintain the biological function of SLC4A7 support a model in which SLC4A7-mediated bicarbonate import into the cytoplasm is crucial for net acid extrusion and maintenance of cytoplasmic pH during phagocytosis. Acidification of the cytoplasm, observed upon SLC4A7 loss, results in impaired acidification of phagosomes and reduced bactericidal capacity (Coakley et al., 2002; Grinstein et al., 1991; Morgan et al., 2009; Hackam et al., 1999).

Phagocytosis, a defining function of macrophages, is a fundamental process throughout macrophage-mediated immunity, starting from the first-line host defense against invading pathogens to removal of apoptotic cell debris associated with tissue remodeling and homeostasis. Given that SLC4A7 affected intracellular bacterial killing or intracellular survival of acid-sensitive and acid-dependent bacteria, respectively, we assume that a wide range of pathogens, including also viruses or parasites, may be affected by SLC4A7 function. This opens the possibility of modulating the activity of this transporter as anti-infective strategy (Huynh and Grinstein, 2007; Ip et al., 2010; Tranchemontagne et al., 2016; Wieland et al., 2004). Failure to remove dead cells and debris in SLC4A7 deficiency may lead to prolonged inflammatory conditions, which in turn may be associated with different pathologies, including cancer, extending the relevance of SLC4A7 function beyond infectious diseases (Novak and Thorp, 2013; Szondy et al., 2014).

Irrespectively, the unequivocal role of SLC4A7 in phagosome acidification of macrophages is surprising, as the protein had not been previously associated with any immune function (Boedtkjer et al., 2011a; Chen et al., 2012; Lopez et al., 2005).

This initial study will clearly have to be followed by animal studies, empowered, for example, by available knockout mice illuminating the role of SLC4A7 in animal physiology (Boedtkjer et al., 2011b; Lee et al., 2016). Nonetheless, the fundamental contribution of SLC4A7 to phagosome acidification is another case where a member of the SLC family is discovered to regulate fundamental biological processes (Rebsamen et al., 2015; Rusu et al., 2017; Wang et al., 2015b). The approach described here, employing a CRISPR/Cas9 library focusing on the entire SLC family allowing for analyses with high coverage and great statistical power, is likely to be instrumental in the uncovering of more roles for this still poorly characterized class of drug targets (César-Razquin et al., 2015; Lin et al., 2015).

STAR★METHODS

Detailed methods are provided in the online version of this paper and include the following:

- KEY RESOURCES TABLE
- CONTACT FOR REAGENT AND RESOURCE SHARING
- EXPERIMENTAL MODEL AND SUBJECT DETAILS
    - Cell Culture
    - Primary Cells and Monocyte to Macrophage Differentiation
    - Culture and Preparation of Bacteria
- METHOD DETAILS
    - SLC-Focused CRISPR/Cas9 Screen
    - Analysis of CRISPR Screens

CellPress

- ○ Phagocytosis Assay
- ○ Flow Cytometry
- ○ Live-Cell Measurements of Cytosolic and Phagosomal pH
- ○ Immunofluorescence
- ○ Cell Lysis and Immunoblotting
- ○ DNA Plasmids and Cloning
- ○ Lentivirus-Mediated DNA Transfer
- ○ Gentamicin Protection Assay
- ● QUANTIFICATION AND STATISTICAL ANALYSIS
- ● DATA AND SOFTWARE AVAILABILITY

SUPPLEMENTAL INFORMATION

Supplemental Information includes two figures, one table, one video, and one data file and can be found with this article online at https://doi.org/10.1016/j.chom.2018.04.013.

ACKNOWLEDGMENTS

We thank J. Praetorius (Aarhus University, Denmark) and J. Casey (University of Alberta, Canada) for providing serum from anti-SLC4A7 immunized mice. We thank N. Sedlyarova, G.I. Vladimer, T. Pemovska, and D. Lackner for assistance, scientific discussion, and critical reading of the manuscript. CeMM and the Superti-Furga laboratory are supported by the Austrian Academy of Sciences (G.S.-F. and I.S.). We acknowledge receipt of third-party funds from the Austrian Science Fund (FWF I2192-B22 ERASE, V.S.; FWF F4711-B20 Myeloid Neoplasms, J.W.B. and F.K.; FWF P29250-B30 VITRA, E.G.; FWF W1205 DK CCHD, A.M.), the European Research Council (ERC AdG 695214 GameofGates, P.E., U.G., and M.R.), the European Commission (Marie Skłodowska-Curie Action Fellowship 661491, E.G.), the European Molecular Biology Organization (EMBO Long-Term Fellowship ALTF245–2017, R.E.), and Boehringer Ingelheim (Research Collaboration Agreement BI-CeMM 238114, L.X.H. and M.R.).

AUTHOR CONTRIBUTIONS

Conceptualization, G.S.-F., V.S., and R.E.; Methodology, G.S.-F., V.S., R.E., P.K., P.N.-H., and N.D.; Investigation, V.S., R.E., E.G., P.E., I.S., A.M., F.K., J.W.B., L.X.H., P.K., P.N.-H., and M.R.; Formal Analysis, V.S. and U.G.; Data Curation, V.S. and U.G.; Writing – Original Draft, R.E., V.S., and G.S.-F.; Writing – Review & Editing, R.E., V.S., and G.S.-F.; Visualization, R.E. and V.S.; Project Administration, V.S. and G.S.-F.; Funding Acquisition, G.S.-F.; Supervision, G.S.-F.

DECLARATION OF INTERESTS

The Superti-Furga laboratory at CeMM has an active research collaboration with Boehringer Ingelheim (Research Collaboration Agreement BICeMM 238114).

Received: October 25, 2017
Revised: March 14, 2018
Accepted: April 19, 2018
Published: May 17, 2018

REFERENCES

Arakawa, T., Kobayashi-Yurugi, T., Alguel, Y., Iwanari, H., Hatae, H., Iwata, M., Abe, Y., Hino, T., Ikeda-Suno, C., Kuma, H., et al. (2015). Crystal structure of the anion exchanger domain of human erythrocyte band 3. Science *350*, 680–684.

Artyomov, M.N., Sergushichev, A., and Schilling, J.D. (2016). Integrating immunometabolism and macrophage diversity. Semin. Immunol. *28*, 417–424.

Barneaud-Rocca, D., Etchebest, C., and Guizouarn, H. (2013). Structural model of the anion exchanger 1 (SLC4A1) and identification of transmembrane segments forming the transport site. J. Biol. Chem. *288*, 26372–26384.

Blomen, V.A., Majek, P., Jae, L.T., Bigenzahn, J.W., Nieuwenhuis, J., Staring, J., Sacco, R., van Diemen, F.R., Olk, N., Stukalov, A., et al. (2015). Gene essentiality and synthetic lethality in haploid human cells. Science *350*, 1092–1096.

Boedtkjer, E., Praetorius, J., Matchkov, V.V., Stankevicius, E., Mogensen, S., Füchtbauer, A.C., Simonsen, U., Füchtbauer, E.-M., and Aalkjaer, C. (2011a). Disruption of Na^+,HCO_3^- cotransporter NBCn1 (slc4a7) inhibits no-mediated vasorelaxation, smooth muscle Ca^{2+} sensitivity, and hypertension development in miceclinical perspective. Circulation *124*, 1819–1829.

Boedtkjer, E., Praetorius, J., Matchkov, V.V., Stankevicius, E., Mogensen, S., Füchtbauer, A.C., Simonsen, U., Füchtbauer, E.-M., and Aalkjaer, C. (2011b). Disruption of Na^+,HCO_3^- cotransporter NBCn1 (slc4a7) inhibits NO-mediated vasorelaxation, smooth muscle Ca^{2+} sensitivity, and hypertension development in mice. Circulation *124*, 1819–1829.

Bonar, P., Schneider, H.-P., Becker, H.M., Deitmer, J.W., and Casey, J.R. (2013). Three-dimensional model for the human Cl^-/HCO_3^- exchanger, AE1, by homology to the *E. coli* ClC protein. J. Mol. Biol. *425*, 2591–2608.

Buck, M.D., Sowell, R.T., Kaech, S.M., and Pearce, E.L. (2017). Metabolic instruction of immunity. Cell *169*, 570–586.

César-Razquin, A., Snijder, B., Frappier-Brinton, T., Isserlin, R., Gyimesi, G., Bai, X., Reithmeier, R.A., Hepworth, D., Hediger, M.A., Edwards, A.M., et al. (2015). A call for systematic research on solute carriers. Cell *162*, 478–487.

Chen, M., Praetorius, J., Zheng, W., Xiao, F., Riederer, B., Singh, A.K., Stieger, N., Wang, J., Shull, G.E., Aalkjaer, C., et al. (2012). The electroneutral Na^+:HCO_3^- cotransporter NBCn1 is a major pH_i regulator in murine duodenum. J. Physiol. *590*, 3317–3333.

Chernova, M.N., Jiang, L., Crest, M., Hand, M., Vandorpe, D.H., Strange, K., and Alper, S.L. (1997). Electrogenic sulfate/chloride exchange in *Xenopus* oocytes mediated by murine AE1 E699Q. J. Gen. Physiol. *109*, 345–360.

Coakley, R.J., Taggart, C., McElvaney, N.G., and O'Neill, S.J. (2002). Cytosolic pH and the inflammatory microenvironment modulate cell death in human neutrophils after phagocytosis. Blood *100*, 3383–3391.

Colas, C., Menezes, S., Gutiérrez-Martínez, E., Péan, C.B., Dionne, M.S., and Guermonprez, P. (2014). An improved flow cytometry assay to monitor phagosome acidification. J. Immunol. Methods *412*, 1–13.

Cooper, D.S., Lee, H.J., Yang, H.S., Kippen, J., Yun, C.C., and Choi, I. (2006). The electroneutral sodium/bicarbonate cotransporter containing an amino terminal 123-amino-acid cassette is expressed predominantly in the heart. J. Biomed. Sci. *13*, 593–595.

Damkier, H.H., Nielsen, S., and Praetorius, J. (2005). An anti-NH2-terminal antibody localizes NBCn1 to heart endothelia and skeletal and vascular smooth muscle cells. Am. J. Physiol. Heart Circ. Physiol. *290*, H172–H180.

Damkier, H.H., Nielsen, S., and Praetorius, J. (2007). Molecular expression of SLC4-derived Na^+-dependent anion transporters in selected human tissues. Am. J. Physiol. Regul. Integr. Comp. Physiol. *293*, R2136–R2146.

Elsinghorst, E.A. (1994). Measurement of invasion by gentamicin resistance. Methods Enzymol. *236*, 405–420.

Fine, N., Barzilay, O., and Glogauer, M. (2017). Analysis of human and mouse neutrophil phagocytosis by flow cytometry. In Methods in Molecular Biology (Clifton), pp. 17–24.

Flannagan, R., Heit, B., and Heinrichs, D. (2015). Antimicrobial mechanisms of macrophages and the immune evasion strategies of *Staphylococcus aureus*. Pathogens *4*, 826–868.

Gratz, N., Siller, M., Schaljo, B., Pirzada, Z.A., Gattermeier, I., Vojtek, I., Kirschning, C.J., Wagner, H., Akira, S., Charpentier, E., et al. (2008). Group A *Streptococcus* activates type I interferon production and MyD88-dependent signaling without involvement of TLR2, TLR4, and TLR9. J. Biol. Chem. *283*, 19879–19887.

Grinstein, S., Swallow, C.J., and Rotstein, O.D. (1991). Regulation of cytoplasmic pH in phagocytic cell function and dysfunction. Clin. Biochem. *24*, 241–247.

Hackam, D., Rotstein, O., and Grinstein, S. (1999). Phagosomal acidification mechanisms and functional significance. In Phagocytosis: The Host (JAI), pp. 299–319.

Huynh, K.K., and Grinstein, S. (2007). Regulation of vacuolar pH and its modulation by some microbial species. Microbiol. Mol. Biol. Rev. *71*, 452–462.

Ip, W.K.E., Sokolovska, A., Charriere, G.M., Boyer, L., Dejardin, S., Cappillino, M.P., Yantosca, L.M., Takahashi, K., Moore, K.J., Lacy-Hulbert, A., et al. (2010). Phagocytosis and phagosome acidification are required for pathogen processing and MyD88-dependent responses to *Staphylococcus aureus*. J. Immunol. *184*, 7071–7081.

Joung, J., Konermann, S., Gootenberg, J.S., Abudayyeh, O.O., Platt, R.J., Brigham, M.D., Sanjana, N.E., and Zhang, F. (2017). Genome-scale CRISPR-Cas9 knockout and transcriptional activation screening. Nat. Protoc. *12*, 828–863.

Kinchen, J.M., and Ravichandran, K.S. (2008). Phagosome maturation: going through the acid test. Nat. Rev. Mol. Cell Biol. *9*, 781–795.

Lee, H.J., Kwon, M.H., Lee, S., Hall, R.A., Yun, C.C., and Choi, I. (2014). Systematic family-wide analysis of sodium bicarbonate cotransporter NBCn1/SLC4A7 interactions with PDZ scaffold proteins. Physiol. Rep. *2*, e12016.

Lee, S., Axelsen, T.V., Andersen, A.P., Vahl, P., Pedersen, S.F., and Boedtkjer, E. (2016). Disrupting Na^+,HCO_3^--cotransporter NBCn1 (Slc4a7) delays murine breast cancer development. Oncogene *35*, 2112–2122.

Lin, L., Yee, S.W., Kim, R.B., and Giacomini, K.M. (2015). SLC transporters as therapeutic targets: emerging opportunities. Nat. Rev. Drug Discov. *14*, 543–560.

Liu, M.Y., and Wu, M.C. (1992). Induction of human monocyte cell line U937 differentiation and CSF-1 production by phorbol ester. Exp. Hematol. *20*, 974–979.

Liu, Y., Qin, X., Wang, D.-K., Guo, Y.-M., Gill, H.S., Morris, N., Parker, M.D., Chen, L.-M., and Boron, W.F. (2013). Effects of optional structural elements, including two alternative amino termini and a new splicing cassette IV, on the function of the sodium-bicarbonate cotransporter NBCn1 (SLC4A7). J. Physiol. *591*, 4983–5004.

Liu, Y., Yang, J., and Chen, L.-M. (2015). Structure and function of SLC4 family HCO_3^- transporters. Front. Physiol. *6*, 355.

Loiselle, F.B., Jaschke, P., and Casey, J.R. (2003). Structural and functional characterization of the human NBC3 sodium/bicarbonate co-transporter carboxyl-terminal cytoplasmic domain. Mol. Membr. Biol. *20*, 307–317.

Lopez, I.A., Acuna, D., Galbraith, G., Bok, D., Ishiyama, A., Liu, W., and Kurtz, I. (2005). Time course of auditory impairment in mice lacking the electroneutral sodium bicarbonate cotransporter NBC3 (slc4a7). Dev. Brain Res. *160*, 63–77.

Love, M.I., Huber, W., and Anders, S. (2014). Moderated estimation of fold change and dispersion for RNA-seq data with DESeq2. Genome Biol. *15*, 550.

Martinez, F.O., Gordon, S., Locati, M., and Mantovani, A. (2006). Transcriptional profiling of the human monocyte-to-macrophage differentiation and polarization: new molecules and patterns of gene expression. J. Immunol. *177*, 7303–7311.

Morgan, D., Capasso, M., Musset, B., Cherny, V.V., Ríos, E., Dyer, M.J.S., and DeCoursey, T.E. (2009). Voltage-gated proton channels maintain pH in human neutrophils during phagocytosis. Proc. Natl. Acad. Sci. USA *106*, 18022–18027.

Novak, M.L., and Thorp, E.B. (2013). Shedding light on impaired efferocytosis and nonresolving inflammation. Circ. Res. *113*, 9–12.

Nunes, P., Guido, D., and Demaurex, N. (2015). Measuring phagosome pH by ratiometric fluorescence microscopy. J. Vis. Exp. https://doi.org/10.3791/53402.

O'Neill, L.A.J., and Pearce, E.J. (2016). Immunometabolism governs dendritic cell and macrophage function. J. Exp. Med. *213*, 15–23.

Parker, M.D., and Boron, W.F. (2013). The divergence, actions, roles, and relatives of sodium-coupled bicarbonate transporters. Physiol. Rev. *93*, 803–959.

Pushkin, A., Abuladze, N., Lee, I., Newman, D., Hwang, J., and Kurtz, I. (1999). Cloning, tissue distribution, genomic organization, and functional characterization of NBC3, a new member of the sodium bicarbonate cotransporter family. J. Biol. Chem. *274*, 16569–16575.

Rebsamen, M., Pochini, L., Stasyk, T., de Araújo, M.E.G., Galluccio, M., Kandasamy, R.K., Snijder, B., Fauster, A., Rudashevskaya, E.L., Bruckner, M., et al. (2015). SLC38A9 is a component of the lysosomal amino acid sensing machinery that controls mTORC1. Nature *519*, 477–481.

Richter, E., Ventz, K., Harms, M., Mostertz, J., and Hochgräfe, F. (2016). Induction of macrophage function in human THP-1 cells is associated with rewiring of MAPK signaling and activation of MAP3K7 (TAK1) protein kinase. Front. Cell Dev. Biol. *4*, 21.

Riihonen, R., Nielsen, S., Väänänen, H.K., Laitala-Leinonen, T., and Kwon, T.-H. (2010). Degradation of hydroxyapatite in vivo and in vitro requires osteoclastic sodium-bicarbonate co-transporter NBCn1. Matrix Biol. *29*, 287–294.

Romero, M.F., Chen, A.P., Parker, M.D., and Boron, W.F. (2013). The SLC4 family of bicarbonate (HCO_3^-) transporters. Mol. Aspects Med. *34*, 159–182.

Rusu, V., Hoch, E., Mercader, J.M., Tenen, D.E., Gymrek, M., Hartigan, C.R., DeRan, M., von Grotthuss, M., Fontanillas, P., Spooner, A., et al. (2017). Type 2 diabetes variants disrupt function of SLC16A11 through two distinct mechanisms. Cell *170*, 199–212.e20.

Sahoo, S., Aurich, M., Jonsson, J., and Thiele, I. (2014). Membrane transporters in a human genome-scale metabolic knowledgebase and their implications for disease. Front. Physiol. *5*, 91.

Sanjana, N.E., Shalem, O., and Zhang, F. (2014). Improved vectors and genome-wide libraries for CRISPR screening. Nat. Methods *11*, 783–784.

Sergushichev, A. (2016). An algorithm for fast preranked gene set enrichment analysis using cumulative statistic calculation. bioRxiv. https://doi.org/10.1101/060012.

Shapiro, H., Lutaty, A., and Ariel, A. (2011). Macrophages, meta-inflammation, and immuno-metabolism. ScientificWorldJournal *11*, 2509–2529.

Soldati, T., Rancano, C., Geissler, H., and Pfeffer, S.R. (1995). Rab7 and Rab9 are recruited onto late endosomes by biochemically distinguishable processes. J. Biol. Chem. *270*, 25541–25548.

Subramanian, A., Tamayo, P., Mootha, V.K., Mukherjee, S., Ebert, B.L., Gillette, M.A., Paulovich, A., Pomeroy, S.L., Golub, T.R., Lander, E.S., et al. (2005). Gene set enrichment analysis: a knowledge-based approach for interpreting genome-wide expression profiles. Proc. Natl. Acad. Sci. USA *102*, 15545–15550.

Surewaard, B.G.J., and Kubes, P. (2017). Measurement of bacterial capture and phagosome maturation of Kupffer cells by intravital microscopy. Methods *128*, 12–19.

Swallow, C.J., Grinstein, S., Sudsbury, R.A., and Rotstein, O.D. (1991). Cytoplasmic pH regulation in monocytes and macrophages: mechanisms and functional implications. Clin. Invest. Med. *14*, 367–378.

Szondy, Z., Garabuczi, É., Joós, G., Tsay, G.J., and Sarang, Z. (2014). Impaired clearance of apoptotic cells in chronic inflammatory diseases: therapeutic implications. Front. Immunol. *5*, 354.

Takahashi, D., Fujihara, M., Miyazaki, T., Matsubayashi, K., Sato, S., Azuma, H., Kato, T., Kino, S., Ikeda, H., Takamoto, S., et al. (2017). Flow cytometric quantitation of platelet phagocytosis by monocytes using a pH-sensitive dye, pHrodo-SE. J. Immunol. Methods *447*, 57–64.

Tranchemontagne, Z.R., Camire, R.B., O'Donnell, V.J., Baugh, J., and Burkholder, K.M. (2016). *Staphylococcus aureus* strain USA300 perturbs acquisition of lysosomal enzymes and requires phagosomal acidification for survival inside macrophages. Infect. Immun. *84*, 241–253.

Via, L.E., Deretic, D., Ulmer, R.J., Hibler, N.S., Huber, L.A., and Deretic, V. (1997). Arrest of mycobacterial phagosome maturation is caused by a block in vesicle fusion between stages controlled by rab5 and rab7. J. Biol. Chem. *272*, 13326–13331.

Wahl, L.M., Smith, P.D., Wahl, L.M., and Smith, P.D. (2001). Isolation of monocyte/macrophage populations. Curr. Protoc. Immunol. https://doi.org/10.1002/0471142735.im0706s16.

Wang, D.-K., Liu, Y., Myers, E.J., Guo, Y.-M., Xie, Z.-D., Jiang, D.-Z., Li, J.-M., Yang, J., Liu, M., Parker, M.D., et al. (2015a). Effects of Nt-truncation and

coexpression of isolated Nt domains on the membrane trafficking of electroneutral Na^{+}/HCO_3^{-} cotransporters. Sci. Rep. *5*, 12241.

Wang, S., Tsun, Z.-Y., Wolfson, R.L., Shen, K., Wyant, G.A., Plovanich, M.E., Yuan, E.D., Jones, T.D., Chantranupong, L., Comb, W., et al. (2015b). Lysosomal amino acid transporter SLC38A9 signals arginine sufficiency to mTORC1. Science *347*, 188–194.

Wieland, H., Goetz, F., and Neumeister, B. (2004). Phagosomal acidification is not a prerequisite for intracellular multiplication of *Legionella pneumophila* in human monocytes. J. Infect. Dis. *189*, 1610–1614.

Yang, J., Yan, R., Roy, A., Xu, D., Poisson, J., and Zhang, Y. (2015). The I-TASSER Suite: protein structure and function prediction. Nat. Methods *12*, 7–8.

STAR★METHODS

KEY RESOURCES TABLE

REAGENT or RESOURCE	SOURCE	IDENTIFIER
Antibodies		
Anti-SLC4A7, rabbit polyclonal	Abcam	Cat#82335; RRID: AB_10672662
Anti-SLC4A7, serum from rabbit immunized with the SLC4A7 N-terminal peptide MEADGAGEQMRPLLTRGPDE	Gift from Prof. J. Praetorius (Aarhus University, Denmark)	(Damkier et al., 2005)
Anti-HA, rabbit monoclonal	Cell signaling	Cat#3724; RRID: AB_1549585
Anti-pan actin, rabbit polyclonal	Cytoskeleton	Cat#AAN01; RRID: AB_10708070
Anti-LAMP1, mouse monoclonal	Abcam	Cat#25630; RRID: AB_470708
Goat anti-mouse IgG, Alexa-Fluor 488 coupled	Life Technologies	Cat#A-11001; RRID: AB_2534069
Donkey anti-rabbit IgG, Cy5 coupled	Jackson ImmunoResearch	Cat#711-175-152; RRID: AB_2340607
Goat anti-rabbit IgG, Peroxidase-conjugated	Jackson ImmunoResearch	Cat#111-035-003; RRID: AB_2313567
Goat anti-mouse IgG, Peroxidase-conjugated	Jackson ImmunoResearch	Cat#115-035-003; RRID: AB_10015289
Bacterial and Virus Strains		
Escherichia coli DH5-alpha	New England Biolabs	Cat#C2987
Streptococcus pyogenes ΔSLO	N/A	(Gratz et al., 2008)
Staphylococcus carnosus Schleifer and Fischer	DSMZ	Cat# 20501
Staphylococcus aureus Newman	Gift from Arsanis Biosciences GmbH to PK	N/A
Staphylococcus aureus USA300	Gift from Prof. S. Knapp (CeMM, Vienna, Austria)	N/A
Chemicals, Peptides, and Recombinant Proteins		
Fluoresbrite YG Carboxylate Microspheres 1.75µm	Polyscience	Cat#17687-5
pHrodo Red, succinimidyl ester (pHrodo Red, SE)	Thermo Fisher Scientific	Cat#P36600
PMA, Phorbol 12-myristate 13-acetate	Sigma-Aldrich	Cat#P8139
Restriction enzyme BsmBI	New England Biolabs	Cat#R0580
BCECF AM	Thermo Fisher Scientific	Cat# B1170
Experimental Models: Cell Lines		
THP-1	ATCC	TIB-202
U937	Gift from Prof. P. Valent, (Medical University Vienna, Austria)	N/A
HEK293T	DSMZ	ACC 635
Oligonucleotides		
See Table S1 for oligonucleotides used to clone sgRNAs	This paper, Sigma	N/A
Recombinant DNA		
lentiCRISPR v2	Addgene	Cat#52961
psPAX2	Addgene	Cat#12260
pMD2.G	Addgene	Cat#12259
pDONR221	Thermo Fisher Scientific	12536017
Codon optimized SLC4A7 isoform 1 and 6 cDNA	GenScript	N/A
Software and Algorithms		
R (3.4.0)	https://www.r-project.org	N/A
Rstudio (1.0.143)	https://www.rstudio.com	N/A
Bioconductor (3.5)	https://bioconductor.org/	N/A
DESeq2 (1.16.1)	https://bioconductor.org/	N/A
Fgsea (1.2.1)	https://bioconductor.org/	N/A

(*Continued on next page*)

Continued		
REAGENT or RESOURCE	SOURCE	IDENTIFIER
ggplot2 (2.2.1)	https://cran.r-project.org/	N/A
Drc	https://CRAN.R-project.org/package=drc	N/A
Other		
RPMI Medium 1640 (1x)	Gibco	Cat#21875-034
DMEM Medium (1x)	Gibco	Cat# 11965-084
DPBS (1x)	Gibco	Cat#14190-094
Fetal Bovine Serum	Gibco	Cat#10270
Pen Strep	Gibco	Cat#15140-122
IC Fixation buffer	eBioscience	Cat#00-8222-49
ProLong Gold Antifade Mountant	Thermo Fisher	Cat#P10144
cOmplete Protease Inhibitor Tablets	Roche	Cat#04693159001
Protein Assay Dye Reagent Concentrate	Biorad	Cat#5000006
Nitrocellulose membranes	Amersham	Cat#10600002
Pierce ECL Western Blotting Substrate	Thermo Scientific	Cat#32106
PolyFect	Qiagen	Cat#301105
QuikChange II Site Directed Mutagenesis Kit	Agilent	Cat# 200523

CONTACT FOR REAGENT AND RESOURCE SHARING

Further information and requests for reagents may be directed to and will be fulfilled by the Lead Contact, Giulio Superti-Furga (gsuperti@cemm.oeaw.ac.at).

EXPERIMENTAL MODEL AND SUBJECT DETAILS

Cell Culture

U937 cells were a gift from Prof. Peter Valent of the Medical University of Vienna, THP-1 cells were purchased from ATCC. Cell lines were tested for mycoplasma with regular intervals. STR profiling was performed for all cell lines to confirm identity.

Cells were cultured in RPMI 1640 supplemented with 10% fetal bovine serum and penicillin/streptomycin with 5% CO_2 at 37°C. U937 cells were maintained at a density of $0.1x10^6$ – $1x10^6$ cells/ml; THP-1 cells were maintained at $0.5x10^6$ – $2x10^6$ cells/ml.

Primary Cells and Monocyte to Macrophage Differentiation

Human monocytes were isolated from healthy donor peripheral blood buffy coats (obtained from the Austrian Red Cross) by adherence to plastic for 3 h in serum-free RPMI medium (Wahl et al., 2001). Monocytes were then differentiated to macrophages for 7 days in RPMI supplemented with 100 ng/ml M-CSF, 10% fetal bovine serum and penicillin/streptomycin. Macrophage polarization was achieved by incubating cells for 18 h in medium with IFNγ (20 ng/ml) and LPS (100 ng/ml) for induction of M1 phenotype or IL-4 (20 ng/ml) for induction of M2 phenotype (Martinez et al., 2006).

Monocyte to macrophage differentiation of THP-1 and U937 cells was performed with 10 nM PMA (Sigma) at a cellular density of $1x10^6$ cells/ml for 48 h followed by washing and an additional 24 h resting period.

Culture and Preparation of Bacteria

Escherichia coli (K12) were inoculated from overnight culture into fresh LB media and grown till mid-log phase (OD_{600} = 0.5). Bacteria were washed in PBS and resuspended to the concentration of $1x10^9$ cfu/ml.

Staphylococcus carnosus (Schleifer and Fisher), *Staphylococcus aureus* (USA300, Newman) were plated on trypcase soy agar with 5% sheep blood from cryo stock and grown for 16 h at 37°C. Single colonies were inoculated in 30 ml of brain heart infusion media (BHI) and grown till reaching the stationary phase (16 h). Overnight cultures were diluted in 50 ml of BHI and grown till mid-log phase. Bacteria were washed 2 times in PBS and resuspended at a concentration of $1x10^9$ cfu/ml.

Streptococcus pyogenes was inoculated in 5 ml of Todd Hewitt Broth with 0.5% yeast (THY) extract and grown at 37°C in atmosphere with 5% CO_2 for 16 h. Overnight cultures were diluted in 30 ml of THY and grown till reaching mid-log phase. Bacteria were washed 2 times in PBS and resuspended at the concentration of $1x10^9$ cfu/ml.

METHOD DETAILS

SLC-Focused CRISPR/Cas9 Screen

The SLC KO CRISPR/Cas9 library used will be described in detail in Girardi et al. (in preparation). Briefly, a CRISPR/Cas9 library targeting 391 SLC genes with six sgRNAs per gene, together with a set of 120 sgRNAs targeting 20 genes essential in KBM7 and HAP1 cells (Blomen et al., 2015) and a set of 120 non-targeting sgRNAs was cloned by Gibson cloning in the lentiCRISPRv2 lentiviral vector. Viral particles were generated by transient transfection of low passage, subconfluent HEK293T cells with the SLC-targeting library and the packaging plasmids psPAX2 and pMD2.G using PolyFect. After 24 h the media was changed to fresh RPMI media supplemented with 10% FCS and antibiotics. The viral supernatant was collected after 48 h, filtered and stored at -80°C until further use. The supernatant dilution necessary to infect U937 cells at a MOI (multiplicity of infection) of 0.2 – 0.3 was determined by puromycin survival after transduction as described previously (Sanjana et al., 2014). U937 cells were infected in triplicates with the SLC KO library at high coverage (1000x) and after selection for 7 days with puromycin (2 μg/ml) an initial sample was collected to control for library composition. Cells were differentiated with PMA (10 nM) to macrophage-like phenotype. Phagocytosis assays were performed with dual-color opsonized latex beads as described in the "Phagocytosis assay" section. Phagocytosis positive (PhagoLate) and phagocytosis negative (PhagoNeg) populations were sorted using BD FACS Aria II, with at least 3 million cells per replicate and population. Genomic DNA was extracted using the DNAeasy kit (QIAGEN) and the cassettes containing the sgRNA sequence were amplified with one round of PCR following the procedure described in Joung et al. (2017). The amplified samples were sequenced on a HiSeq2000 (Illumina) at the Biomedical Sequencing Facility (BSF at CeMM, https://biomedical-sequencing.at), followed by processing with a custom analysis pipeline (see next section).

Analysis of CRISPR Screens

Sequences of sgRNAs were extracted from RNA-Seq reads, matched against the original sgRNA library index and counted using an in-house Python script. To compensate for the noise and off-target action of sgRNAs inherent to CRISPR screening approaches, we used a two-step differential abundance analysis. Differential abundance of sgRNAs was estimated with DESeq2 (Love et al., 2014). Subsequenly, sgRNAs were sorted by adjusted p-values and aggregated to genes using Gene Set Enrichment Algorithm (Sergushichev, 2016; Subramanian et al., 2005).

Phagocytosis Assay

Phagocytosis assays were performed as previously described (Colas et al., 2014). Fluoresbrite carboxylated 1.75 μM microspheres (YG: 441 nm excitation, 486 nm emission) were opsonized in 20% human male AB serum in PBS for 16 h at 4°C with constant rotation. Subsequently beads were washed twice with PBS and stained with 2 μg/ml pHrodo-Red, SE for 30 min at room temperature with agitation. After washing, beads were resuspended to a final concentration of $1x10^9$ beads/ml.

PMA-differentiated cells (U937 or THP-1) were seeded on 12-well cell culture coated dishes ($1x10^6$ cells/well). Stained beads were added to cells at the indicated MOIs. Dishes were centrifuged at 150 g for 10 min with break set to off. After 30 min incubation, cells were washed three times with warm PBS. Cells were further incubated for the indicated times and then detached by scraping with soft rubber policeman and analyzed by flow cytometry.

Flow Cytometry

All data acquisition was performed using the LSR Fortessa II cytometer interfaced with FACSDiva (BD). FlowJo X software (Tree Star) was used for data analysis and graphical representation.

Live-Cell Measurements of Cytosolic and Phagosomal pH

THP-1 and U937 cells were seeded on imaging dishes (ibidi 35 mm dishes with glass bottom) and differentiated with PMA (10 nM) for 48 h. Differentiated cells were washed twice in HBSS and subsequently loaded with BCECF-AM (5 μM) in HBSS with sodium bicarbonate and 10 mM HEPES for 30 min at 37°C. After washing three times in HBSS, the cells were supplemented with HBSS with 10% FBS. Dual-colored beads (pHrodo and bright blue) or pHrodo-coupled heat-killed S.aureus were added to the cells at an MOI of 10. After spinning at 1000 rpm for 2 min, the cells were further incubated in humidified atmosphere at 37°C and 5% CO_2. Live cell microscopy was performed on a Zeiss Axio Oberver Z1 widefield microscope (at 37°C and in 5% CO_2) at 1 and 6 h after loading the phagosomal cargo. pHrodo and bright blue signal was acquired in the respective channels (RFP/DAPI). BCECF emission at 525 nm was acquired after both excitation at 440 nm and 490 nm respectively. 5 independent z-stacks were acquired per dish, and three independent replicate dishes imaged per condition.

An *in situ* calibration curve was constructed by incubating cells in potassium buffers with 10 μM valinomycin and 10 μM nigericin with ranging pH (4.0 – 10.0).

For image analysis, maximum-projections of z-stacks were generated, background signal was subtracted and signal intensity in respective channels was measured in the respective regions of interest (cytoplasm of cells having undergone phagocytosis; acidified phagosomal particles).

Immunofluorescence

For immunofluorescence, THP-1 cells or U937 cells were seeded on glass coverslips and differentiated with PMA for 48 h. For staining of endogenous SLC4A7, PMA-differentiated control (sgRen) and SLC4A7 knockout (sg1) THP-1 cells were washed in PBS. Fixation buffer was added for 10 min at RT (room temperature), cells were washed in PBS again and ice-cold methanol was added for 10 min at 4°C. Subsequently, cells were blocked in IF buffer (2.5% BSA, 0.5% Tween in PBS) for 30 min and next incubated with anti-SLC4A7 (rabbit) primary antibody (at 1:200 dilution) in IF buffer over night. After washing 3 times, Alexa Fluor 488-coupled anti-rabbit (Life Technologies) was added at a dilution of 1:1000. DAPI was used to counterstain DNA and slides were mounted with ProLong Gold Antifade Mountant.

For localization of SLC4A7 isoform 1, isoform 6, and the respective SLC4A7 mutants, U937 cells expressing Strep-HA-tagged forms of SLC4A7 were subjected to the same fixation procedure and incubated with anti-HA (rabbit) and anti-Lamp1 (mouse) primary antibodies (at a 1:1000 and 1:200 dilution in IF buffer respectively), and Alexa Fluor 488-coupled anti-mouse (Life Technologies) and Cy5-coupled anti-rabbit (Jackson ImmunoResearch) secondary antibodies respectively.

Images were taken using a laser-scanning confocal microscope LSM700 (Zeiss).

For live-cell microscopy of GFP-SLC4A7 expressing THP-1 cells, these cells were seeded on chambered coverslips (ibidi) and differentiated with PMA (10 nM) for 48 h. Media was replaced by HBSS with 10 mM of HEPES and 10% of FBS, and dual-colored beads (pHrodo and bright blue) or pHrodo-coupled heat-killed S.aureus were added to the cells at an MOI of 10. After spinning at 1000 rpm for 2 min, the cells were further incubated in humidified atmosphere at 37°C and 5% CO_2. Live cell microscopy including time-lapse series was performed on a laser-scanning confocal microscope LSM780 (Zeiss) at 37°C and in 5% CO_2.

Cell Lysis and Immunoblotting

Cell samples were lysed in lysis buffer (NaCl 150 mM, Tris-HCl 50 mM, $MgCl_2$ 5 mM, EDTA 1 mM, NP-40 0.5 %, Glycerol 5 %) to which cOmplete Protease Inhibitor Tablets were added. After lysis in appropriate volumes and 20 min incubation on ice, cellular lysates were centrifuged at 14000 rpm and 4°C for 15 min to separate from cellular debris, protein concentration was measured according to the Bradford method using Protein Assay Dye Reagent Concentrate, and Laemmli buffer was added. The lysates were separated by SDS-PAGE and blotted onto nitrocellulose membranes. After blocking unspecific binding in 5% milk in TBS-T, membranes were incubated with primary antibodies diluted in 5% milk or 5% BSA (bovine serum albumin) in TBS-T. After addition of peroxidase coupled secondary antibodies, immunoblot membranes were developed using the ECL (enhanced chemoluminescence) method.

DNA Plasmids and Cloning

Codon-optimized cDNA for SLC4A7 isoform 1 and isoform 6 was synthesized by GenScript. After sequence verification, both cDNAs were first cloned into the pDONR221 plasmid and then shuttled into a modified pRRL-based lentiviral expression plasmid generated in our lab, which contains an N-terminal Strep-HA tag and a blasticidin resistance cassette. The cDNA of isoform 6 was additionally shuttled into another modified pRRL-based lentiviral expression plasmid generated in our lab, which contains an N-terminal GFP tag and a hygromycin resistance cassette.

The C-terminal deletion mutant of SLC4A7 isoform 6, lacking amino acids 1008-1131, was generated by PCR with respective primers and cloned as described above. For generation of the two SLC4A7 point mutants T549I and D811A, mutagenesis primers were designed using the QuikChange Primer Design tool from Agilent. Mutagenesis was performed using the QuikChange II Site-Directed Mutagenesis Kit (Agilent), following the instructions of the manufacturer. After sequence verification, the cDNAs were shuttled into the pRRL plasmid described above.

sgRNAs were cloned into Bsm BI sites of lentiCRISPR v2.

Lentivirus-Mediated DNA Transfer

For lentivirus production, HEK 293T cells were transfected with the sgRNA or expression plasmid, the envelope plasmid pMD2.G and the packaging plasmid psPAX2 using PolyFect. 48 h after transfection, the virus-containing medium was collected and filtered. For infection, THP-1 and U937 cells were plated in 6 well plates, in 2 ml of fresh medium,1 ml of harvested virus and 8 μg/ml protamine sulphate, spin infected at 2000 rpm for 45 min and incubated with the virus-containing supernatant for 24 h. Next day, medium was exchanged. Three days after infection, selection was started by addition of blasticidin (30 μg/ml) or puromycin (2 μg/ml) respectively.

Gentamicin Protection Assay

Gentamycin protection assays were performed as previously described (Elsinghorst, 1994; Richter et al., 2016). Briefly, PMA-differentiated THP-1 cells ($0.75x10^6$ cells per well on 24-well plates) were inoculated with bacteria at an MOI of 10. Plates were centrifuged at 150 g for 5 min with break set to off. After 30 min, cells were washed with PBS 3 times and supplemented with fresh medium containing 100 μg/ml gentamicin to remove or kill extracellular bacteria. After 30 min, cells were washed and supplemented with medium containing 5 μg/ml gentamycin. At consecutive time points, SLC4A7 knock-out and control THP-1 cells were lysed and plated on agar to allow for outgrowth of surviving intracellular bacteria.

QUANTIFICATION AND STATISTICAL ANALYSIS

Exploratory data analysis, visualization, and statistical testing were performed with R-project (http://www.R-project.org) using Rstudio IDE. Pair-wise differences in central tendencies were tested using Welch's t-test or Wilcoxon-Mann-Whitney-Test as indicated in figure legends. Error bars represent 95% confidence intervals, unless indicated otherwise in the figure legends.

DATA AND SOFTWARE AVAILABILITY

sgRNA count data are provided in Data S1.

Cell Host & Microbe, Volume *23*

Supplemental Information

The Bicarbonate Transporter SLC4A7 Plays a Key Role in Macrophage Phagosome Acidification

Vitaly Sedlyarov, Ruth Eichner, Enrico Girardi, Patrick Essletzbichler, Ulrich Goldmann, Paula Nunes-Hasler, Ismet Srndic, Anna Moskovskich, Leonhard X. Heinz, Felix Kartnig, Johannes W. Bigenzahn, Manuele Rebsamen, Pavel Kovarik, Nicolas Demaurex, and Giulio Superti-Furga

Supplementary Figure 1

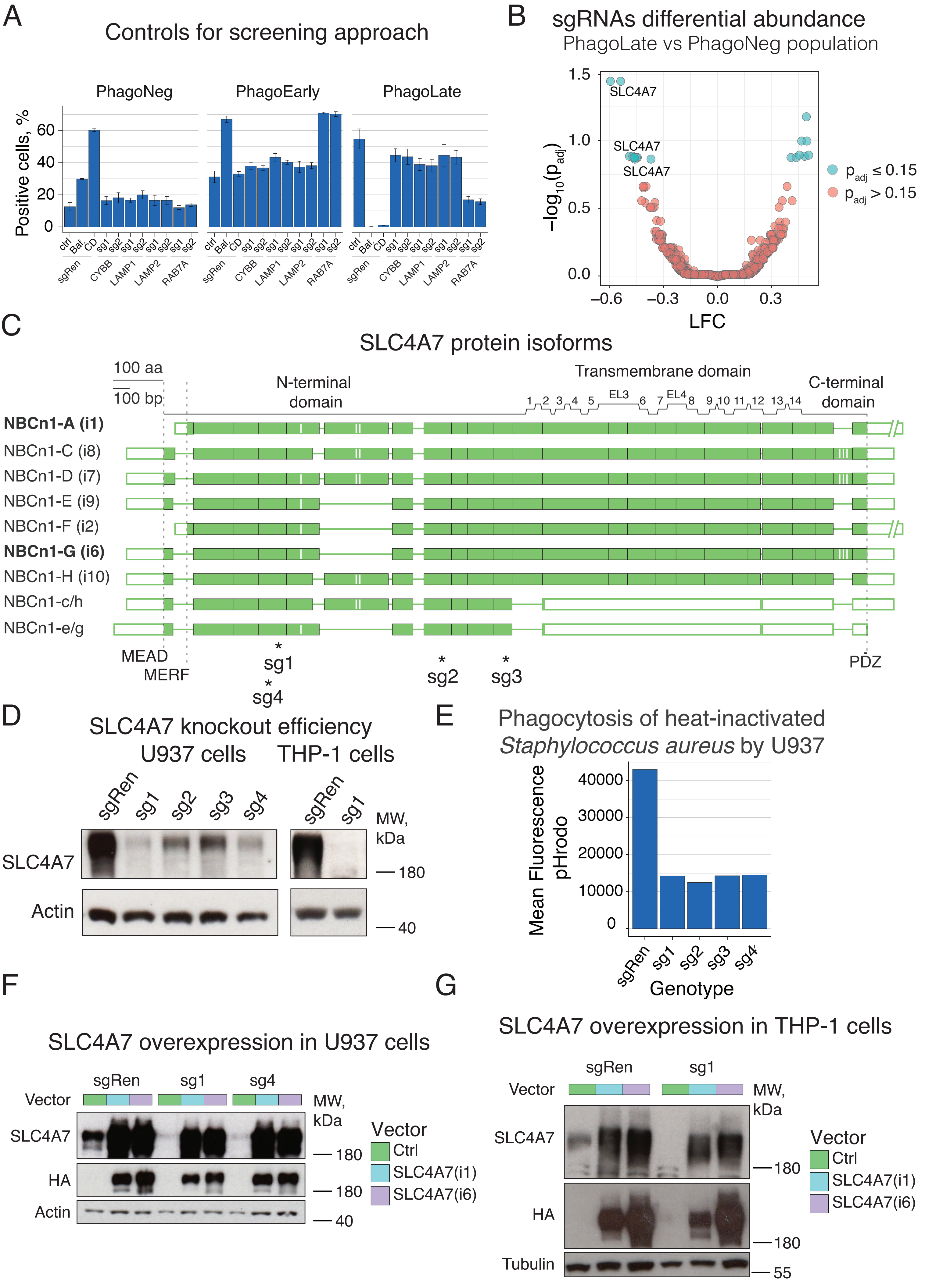

Figure S1. Loss of SLC4A7 impairs phagocytosis and phagosomal acidification. Related to Figure 1.

(A) Phagocytosis assays of U937 cells control (sgRen), treated with Bafilomycin A1 (Baf.) or Cytochalasin D (CD), NOX2 (Cybb), Lamp1, Lamp2 and Rab7a knockouts with 2 independent sgRNAs (sg1, sg2). Bar charts represent mean PhagoLate, PhagoEarly and PhagoNeg fractions as defined in Figure 1B, error bars represent 95 % confidence interval.

(B) Volcano plot showing the statistical significance of differential abundance of sgRNAs in the PhagoLate compared to the PhagoNeg fraction as $-\log_{10}(p_{adj})$ on the y-axis against their $\log_2$ fold-change (LFC) on the x-axis. The color of the dot represents statistically significant differential abundance at an estimated 15 % false discovery rate.

(C) Schematic representation of selected SLC4A7 isoforms. Exons are depicted as green rectangles and transmembrane topology is shown by the profile line above. Transmembrane domains (TMDs) are numbered 1 – 14 and the two larger extracellular loops (EL) are annotated (EL3, EL4). The alternative splicing cassettes (I – III) are shown as numbers within the exons and the two alternative start sites (“MERF” and “MEAD”) are indicated by the dotted lines. The position of the PDZ domain in the C-terminus is indicated by a dotted line. Sites targeted by the selected sgRNAs are marked with asterisks.

(D) Representative immunoblot analysis of PMA-differentiated U937 (left panel) and THP1 (right panel) cells, which were infected with the indicated sgRNA constructs (sg1-sg4) targeting SLC4A7 or control (sgRen). Lysates were probed with antibodies detecting SLC4A7 or actin as loading control.

(E) Phagocytosis assays of SLC4A7 knockout (sg1 – sg4) and control (sgRen) U937

cells with pHrodo-Red stained heat-killed *Staphylococcus aureus* USA300. Bar graphs depict mean fluorescence intensity of pHrodo signal. Increasing pHrodo-Red signal depicts increasing acidification of phagocytosed bacteria.

(F) Representative immunoblot analysis of two independent U937 clones with SLC4A7 knockout (sg1, sg4) or control (sgRen), which were infected with lentiviral expression constructs coding for Strep-HA tagged SLC4A7 isoform 1 (SLC4A7(i1)), isoform 6 (SLC4A7(i6)) or empty vector control (empty). Respective lysates were probed with the indicated antibodies.

(G) Immunoblot analysis with control (sgRen) and SLC4A7 knockout (sg1) THP-1 cells, which were infected with lentiviral expression constructs coding for Strep-HA tagged SLC4A7 isoform1 (SLC4A7-i1), isoform6 (SLC4A7-i6) or empty vector control (Ctrl). Respective lysates were probed with the indicated antibodies.

Supplementary Figure 2

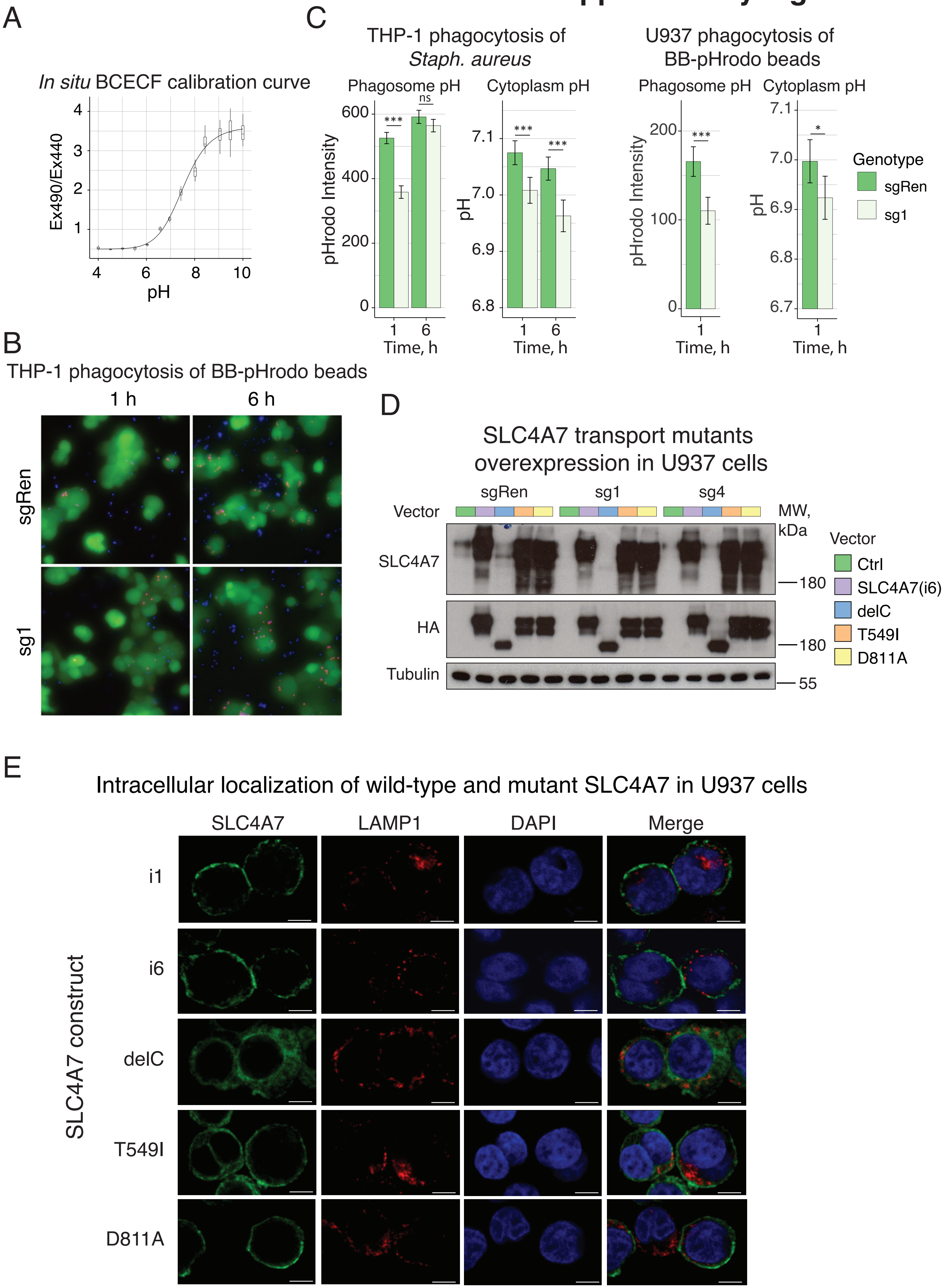

Figure S2. Reconstitution with wildtype but not mutant SLC4A7 rescues the phenotype of SLC4A7 knockout. Related to Figure 2.

(A) The *in situ* calibration curve was constructed by measuring the fluorescence ratio of BCECF emission at 525nm after excitation at 440nm or 490nm. THP-1 cells were equilibrated in potassium buffers with varying pH (4.0 – 10.0) in the presence of 10 µM nigericin and 10 µM valinomycin. Intracellular pH values are represented as box plots. Sigmoid curve was fitted to the data.

(B) Example images of simultaneous cytoplasmic and phagosomal pH measurements in THP-1 cells phagocyting bright blue pHrodo beads. THP-1 cells loaded with BCECF are shown in green (excitation 490 nm, emission 525 nm), dual-coloured (bright blue and pHrodo) beads are shown in blue and red respectively.

(C) Simultaneous measurement of cytoplasmic and phagosomal pH during phagocytosis using live-cell microscopy. Cytoplasmic and phagosomal pH of THP-1 cells loaded with BCECF-AM was measured at 1h and 6 h of pHrodo-labeled heat-killed *Staph aureus* phagocytosis (left panel). Cytoplasmic and phagosomal pH of U937 cells loaded with BCECF-AM was measured at 1h of dual-colored beads phagocytosis (right panel). Incubation and imaging were done at 37°C in 5% CO_2 in HBSS/10 % FCS buffer.

(D) Representative immunoblot analysis of two independent U937 clones with SLC4A7 knockout (sg1, sg4) or control (sgRen), which were infected with lentiviral expression constructs coding for Strep-HA tagged SLC4A7 isoform 6 (SLC4A7(i6)), a C-terminal deletion mutant (delC), two predicted transport mutants (T549I and D811A), or empty vector control (Ctrl). Lysates were probed with the indicated antibodies.

(E) Representative confocal immunofluorescence images of U937 cells infected with

lentiviral expression constructs coding for Strep-HA tagged SLC4A7 isoform 1 (i1), isoform 6 (i6), a C-terminal deletion mutant (delC) or two predicted transport mutants (T549I and D811A). Cells were differentiated with PMA and stained with antibodies to HA (green) and LAMP-1 (red). DNA counterstain with DAPI is depicted in blue. Scale bar is 5 µm.
